# Redirector: Designing Cell Factories by Reconstructing the Metabolic Objective

**DOI:** 10.1371/journal.pcbi.1002882

**Published:** 2013-01-17

**Authors:** Graham Rockwell, Nicholas J. Guido, George M. Church

**Affiliations:** 1 Department of Genetics, Harvard Medical School, Boston, Massachusetts, United States of America; 2 Program in Bioinformatics, Boston University, Boston, Massachusetts, United States of America; University of Virginia, United States of America

## Abstract

Advances in computational metabolic optimization are required to realize the full potential of new *in vivo* metabolic engineering technologies by bridging the gap between computational design and strain development. We present Redirector, a new Flux Balance Analysis-based framework for identifying engineering targets to optimize metabolite production in complex pathways. Previous optimization frameworks have modeled metabolic alterations as directly controlling fluxes by setting particular flux bounds. Redirector develops a more biologically relevant approach, modeling metabolic alterations as changes in the balance of metabolic objectives in the system. This framework iteratively selects enzyme targets, adds the associated reaction fluxes to the metabolic objective, thereby incentivizing flux towards the production of a metabolite of interest. These adjustments to the objective act in competition with cellular growth and represent up-regulation and down-regulation of enzyme mediated reactions. Using the iAF1260 *E. coli* metabolic network model for optimization of fatty acid production as a test case, Redirector generates designs with as many as 39 simultaneous and 111 unique engineering targets. These designs discover proven *in vivo* targets, novel supporting pathways and relevant interdependencies, many of which cannot be predicted by other methods. Redirector is available as open and free software, scalable to computational resources, and powerful enough to find all known enzyme targets for fatty acid production.

## Author Summary

A deeper understanding of biological processes, along with methods in synthetic biology, is driving the frontier of metabolic engineering. In particular, a better representation of cell metabolism will enable the engineering of bacterial strains that can act as factories for valuable biochemical products, from medicines to biofuels. Models which predict the behavior of these complex biological systems enable better engineering design as well as a more comprehensive understanding of fundamental biological principles. Here we develop a new method, called Redirector, for modeling metabolic alterations, and their relationship to cell growth. This method optimizes genetic engineering changes to achieve metabolite production using a new representation of the metabolic impact of genetic manipulation, which is more biologically realistic than existing models. We discover proven and novel engineering targets to improve fatty acid production, correctly predicting how different combinations of genes build upon one another. This work demonstrates that Redirector is a powerful method for designing cell factories and improving our understanding of metabolic systems.

## Introduction

Building a better predictive understanding of genome-scale metabolic networks is critical to fully harnessing the power of bacterial metabolism in general and for designing biofactories in particular. Biofactories have been engineered for a variety of products including pyruvate [1], [2], succinate [3], [4], fatty acid derived biofuels [5], [6], and isoprenoid pathway products [7]–[15]. Recent advances in synthetic biology make it possible to engineer larger numbers of genetic components either by rapid assembly of modular parts [16]–[21] or by multiplexed directed evolution of large sets of specific loci [9], [22]. Effective use of these next generation capabilities requires new computational design tools that can analyze these large sets of metabolic engineering targets for less obvious but highly effective combinations of metabolic alterations, directing metabolic flux towards metabolite overproduction.

While kinetic models have been developed to simulate biological networks [16], [23]–[31], these methods are limited by their computational complexity and their sensitivity to regulatory and kinetic parameters, which largely remain unknown. Meanwhile, using steady-state models with only stoichiometric constraints has proven robust and effective. Flux balance analysis (FBA) models cellular metabolism by imposing theses constraints and optimizing an objective function, such as the production of biomass representing growth and replication of the organism [32]–[34]. Additional constraints [35]–[38], and different forms of the objective function [37], [39] incorporating additional biological information, have been shown to improve the predictive capacity of FBA models for metabolic response to changing conditions and metabolic engineering. However, maximization of biomass is one of the best understood objectives [40] and has proven to be one of the best predictors for cellular behavior resulting from various metabolic alterations [41].

FBA has been used to successfully design production strains by optimizing metabolic alterations such as gene knockouts [42], [43], setting flux levels [44] and the addition of exogenous reactions [45]. These methods use a bilevel optimization framework leveraging FBA in a formulation where an “inner” biomass objective is optimized for growth subject to restrictions set by an “outer” production objective, optimizing a particular metabolite flux. Yet even with these deft applications of FBA modeling, predicting globally optimal metabolic changes is often a computationally difficult problem. In Genetic Design using Local Search (GDLS) [46] we combined bilevel optimization with gene targets mapped to reactions in an iterative local searching algorithm discovering a more complete set of knockout targets. Recent methods for optimizing metabolic alteration targets have gone beyond knockouts and modeled up- and down-regulations [44], [47], [48]. These approaches are strongly dependent on all aspects of the system, natural and engineered, being modeled by flux bounds.

Representing genetic alterations to a metabolic system (specifically up- and down-regulation) as direct changes to flux boundaries presents a number of problems. Firstly, representing metabolic alterations in this way is difficult in the common case when a single enzyme affects many reactions. Imposing different flux bounds on reactions controlled by the same enzyme creates a disconnect with experimental implementation. However, applying the same flux constraints to all reactions controlled by one such enzyme will often fail to produce the desired metabolite. Second, using a limit on one flux is sufficient to control a whole pathway, and all up-stream reactions, in an FBA model. Further, multiple metabolic alterations will often only be as effective as the tightest limit imposed, and metabolic alterations beyond the single strongest will often provide no additional benefit. *In vivo* engineering and kinetic modeling have shown that engineering individual enzymes has a limited potential to change the flux through a metabolic pathway [16], [23]–[27] and have proven the effectiveness of harnessing the cumulative effects of changing many enzymes [9]. Finally, studies in adaptive evolution have shown that metabolic systems will adapt to accommodate or counteract metabolic engineering changes [49]. However, modeling metabolic alteration with flux constraints must be obeyed, hence modeling such adaptation would be difficult.

To address the above issues and in order for metabolic design optimization to reach its full potential, metabolic alterations must be represented in a more biologically relevant way. Hence, we develop a framework modeling metabolic alterations using the FBA objective to represent the balance of resource allocation between growth and metabolite production. Metabolic alterations (up- and down- regulations) are modeled through incentivizing flux changes by adding reaction fluxes to the existing FBA biomass objective with associated positive or negative coefficients. These coefficients determine the relative strengths and direction of the impact the metabolic alterations have on the reaction fluxes. We can then find a set of metabolic alterations optimized for the production of a metabolite of interest, using a bilevel optimization approach. To make this possible, we develop a method by which an optimal set of reactions, grouped by enzyme, can be included in, or excluded from, the metabolic (inner) objective. We maintain the original flux that makes up the biomass function, along with the added incentives on fluxes towards metabolite production in the inner FBA objective, to ensure that selected metabolic alterations toward metabolite production account for cellular growth.

It is important to enable Redirector to discover a growing set of metabolic alterations that work synergistically to drive flux towards production to better overcome growth driven adaptation and regulatory mechanisms. To achieve this, we improve the iterative local search in GDLS [46] to harness the Redirector model of metabolic alterations. During each iteration, the biomass contribution to the inner objective of the bilevel problem is adjusted to drive the discovery of additional metabolic alterations until we have exhausted the set of available enzyme targets. An increased relative strength for the biomass component in the objective necessitates a larger number of metabolic alterations to drive flux towards metabolite production. With these methods we are able to target enzymes affecting many reactions, as well as find targets that work cooperatively, even in linear pathways.

## Results

**Figure 1:**
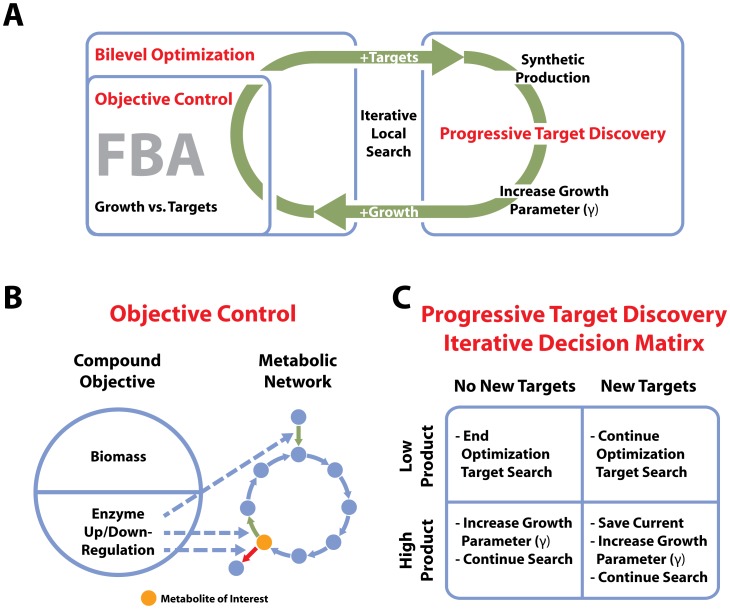
Redirector algorithm. A. Here are the novel aspects of the Redirector algorithm brought together to depict the algorithmic flow. An iterative local search alternates between a bilevel optimization using objective control and the progressive target discovery. Objective control produces enzyme genetic alterations (+targets) and the associated metabolite production level, while the progressive target discovery increases the progressive growth parameter, or γ (+growth), based on the enzyme optimization targets and metabolite production level, from the previous iteration. B. The objective control method involves an FBA objective that includes the biomass (growth) flux as well as a selected set of enzyme associated reaction fluxes, which are up- or down-regulated. An optimized set of enzymes is included in the objective to drive the production of the metabolite of interest. The dotted lines show that an enzyme appearing in the objective incentivizes changes in the associated reaction fluxes. C. The progressive target discovery method adjusts a coefficient on the biomass term, used in objective control, after each iteration of the optimization. Here we show a decision tree for the adjustment of the progressive growth parameter based on the discovery of new targets, and the metabolite production level from the previous iteration.

Figure 1A shows how the Redirector framework brings together new methods to create metabolic engineering designs. The framework takes advantage of an iterative local neighborhood search, as demonstrated in GDLS [46]. This iterative local search method limits the number of new metabolic alteration targets, chosen per iteration of bilevel optimization, to a particular search size (k). At each iteration, the optimization is able to add up to k genetic alteration targets to those from previous iterations. In Redirector, the iterative local optimization cycles between two novel methods. The first is called “objective control”, which finds metabolic engineering targets at the enzyme level and adds them to the objective. The second is a method called “progressive target discovery”, which leverages our use of objective reconstruction, iteratively adjusting the contribution of growth to the objective, driving the discovery of new targets to redirect resources to the production of the metabolite being optimized.

In objective control (Figure 1B) the inner, biomass objective of the bilevel formulation is altered to become a compound objective. This compound objective includes the original biomass reaction, but also allows for metabolic alterations to be represented by adding reaction fluxes to this objective. The bilevel optimization selects metabolic engineering targets with the use of a particular “inclusion” variable that determines the inclusion or exclusion of corresponding reactions to the objective. Optimization for production of a metabolite in the outer objective of the bilevel formulation drives the algorithm to add reactions to the inner objective that will enable production of this metabolite. It is also important to note that these metabolic alterations are carried out on the enzyme level. This means that when an enzyme is chosen as a target, all reactions that this enzyme controls are added to the objective. Enzyme mediated reactions, having been added to the objective, redirect flux towards production of the metabolite. Reactions are added to the objective with a coefficient that determines the level of up-regulation or down-regulation for that reaction, with all reactions that are mediated by a single enzyme having the same coefficient.

Because both the biomass and reactions leading to the production of a metabolite appear in the objective, if a high metabolite production level is achieved, discovery of new targets will only continue after increasing the incentive for growth in the objective. This process incentivizes resources to biomass and necessitates new metabolic alterations to meet the metabolite production goal, dictated by the outer objective of the bilevel optimization. For this reason, we have developed progressive target discovery. After each iteration of bilevel optimization using objective control, the Redirector algorithm checks if there have been new metabolic engineering targets discovered, and also checks the level of production of the metabolite for which we are optimizing. Figure 1C illustrates the decision process that the algorithm uses to determine when to incentivize growth in the objective before the next optimization iteration. If, in the previous iteration, metabolite production is low (below 80% of optimal) and new targets have been found, the optimization continues with no added incentive to growth in the objective. When the metabolite production is high (at or above 80% of optimal), and there are no new targets, Redirector increases a coefficient on the biomass component of the objective, called the progressive growth parameter (γ), and continues the next iteration of optimization. When the production level is high and new targets have been discovered, the algorithm saves the current target set, increases the progressive growth parameter and continues the search. Finally, if the metabolite production is low and there are no new targets, the optimization ends, as it has exhausted target possibilities. Once the search ends, the previously saved set of targets resulting in the highest progressive growth parameter is considered the final design. As a result, this cycle continues until no more metabolic engineering targets can be discovered.

The optimal set of targets to drive metabolite production is in large part determined by their relative weighting in the objective. Reaction fluxes are included in the objective using weights, called redirection coefficients (β), selected from a set of values we call the redirection coefficient library. In this work we focus on three methods we have developed for constructing the coefficient library which we term the flat, power series and sensitivity redirection coefficient libraries (discussed in the Redirection Coefficient Library section in the Supporting Information Text S1). The flat library uses β ∈ (1.0,−1.0) for every reaction, while the power series library uses β ∈ (2^n^,−2^n^) where n≤0, and the sensitivity library is created by performing sensitivity analysis for every reaction on each of the growth and production objectives. The flat library is the simplest approach, giving each reaction equal weight but not allowing further tuning of the targets. The power series library allows for tuning the impact of metabolic targets but is computationally intensive. Finally, the sensitivity library indicates how a reaction flux influences the metabolite production or growth, with only those reactions that directly affect one of these objectives getting a coefficient. Thus, using the sensitivity redirection coefficient leads to a smaller pool of enzymes from which to select targets, and therefore, a less computationally intensive optimization.

To demonstrate metabolic engineering designs produced by the Redirector framework, we focus on the production of the fatty acids, in particular myristoyl-CoA (C14:0-CoA), using the iAF1260 genome-scale *Escherichia coli* MG1655 model [50]. Fatty acids are a well-studied precursor for biofuels [5], [6], like fatty alcohols and fatty acid ethyl esters, and can be targeted specifically by particular thioesterases, thus exported outside the cell. For this work we created a reaction which exports myristoyl-CoA and recycles CoA. This product provides a test of the framework for production of metabolites involving complex pathways. The fatty acid pathways are outside of core metabolism (core metabolism is widely recognized as glycolysis, gluconeogenesis, the Krebs cycle, pentose phosphate pathway, purine and pyrimidine metabolism, and amino acid metabolism) and feature complex enzyme-metabolite relationships, in which the enzymes of both the fatty acid biosynthesis and degradation pathways metabolize different lengths of both saturated and unsaturated fatty acids.

### Redirector Performance

**Figure 2:**
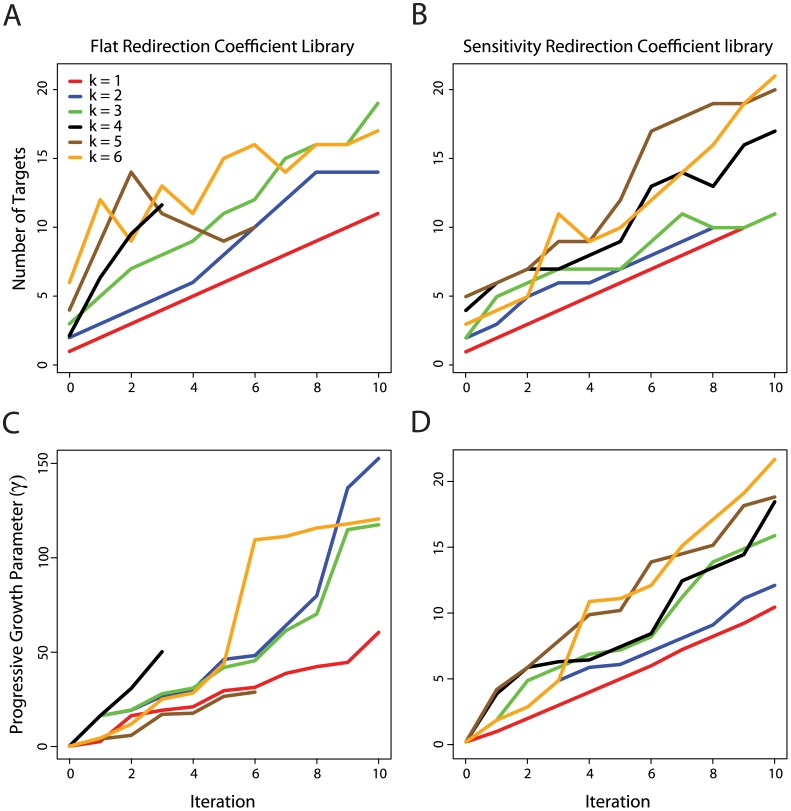
Number of targets and γ vs. iteration for optimization of myristoyl-CoA. *k* indicates the neighborhood size of the search. Red lines represent neighborhood size 1, blue lines are neighborhood size 2, green lines are neighborhood size 3, black lines are neighborhood size 4, brown lines are neighborhood size 5 and orange lines are neighborhood size 6. A. Number of targets vs. iteration for the flat redirection coefficient library. B. Number of targets vs. iteration for the sensitivity redirection coefficient library. C. γ vs. iteration for the flat redirection coefficient library. D. γ vs. iteration for the sensitivity redirection coefficient library.

To provide insight into the progress of design construction using the Redirector Framework, we present Figure 2, which shows the number of targets and progressive growth parameter (γ) discovered by optimizations using both the flat and sensitivity redirection coefficient libraries separately. Different neighborhood search sizes (*k)* were run to 10 iterations optimizing for myristoyl-CoA. Here we allow no tuning of the level of metabolic alteration (tuning of the redirection coefficients and the associated parameter (*s*) are discussed in the Supporting Information Text S1 section Bilevel Optimization Problem). These graphs elucidate the relationship between the progressive growth parameter and the number of targets found, as well as how target number and γ are influenced by the redirection coefficient library.

Figure 2A shows results based on the flat redirection coefficient library, while Figure 2B shows the results for the sensitivity coefficient library. When using larger search sizes, such as k = 5 and k = 6, the framework finds better solutions by ‘backtracking’, removing some previously obtained reactions while adding others. Figure 2A shows that search sizes *k* = 4 and *k* = 5 do not progress beyond iteration 3 and 6 respectively, indicating that no more solutions were found in the allotted search times beyond this point. We observe that searching with different neighborhood sizes allows us to discover designs that overcome locally optimal solutions and the challenging nature of MILP optimizations. Preselecting the redirector coefficient library with a sensitivity analysis improves search performance as indicated by figure 2B where all searches reach 10 iterations.

Figure 2 C and D show the value of the progressive growth parameter used to increase the growth contribution to the objective and produce each design at a particular iteration. The scale of the progressive growth parameter for flat and sensitivity redirection coefficient libraries is different because the growth parameter value needed to drive design discovery is directly dependent on the scale of the values in the redirection coefficient library being used. In these graphs we observe that increasing progressive growth parameter is necessary to drive the discovery of larger sets of targets.

### Design Discovery Overview

To give an overview of the performance of the Redirector method we present Table 1 of design summary statistics. Here, we show the results of performing optimization using the Redirector framework for production of malonyl-CoA, and saturated and mono-unsaturated fatty acids with carbon chain lengths between 14 and 18. Optimizations were performed for neighborhood sizes between 1 and 6, and up to 15 iterations using both the flat redirection coefficient library and sensitivity redirection coefficient library. Table 1 shows that the Redirector method finds 115 targets, on average, for the representative fatty acid objectives listed. The size of the largest individual designs is about a quarter of the total unique targets, indicating that a number of different orthogonal design solutions are being discovered. Different solutions are found due to differing redirection coefficient libraries, which weight reactions differently in the optimization, as well as independent search trajectories taken by different neighborhood search sizes, at each iteration.

**Table 1:** Target totals.

Product	Unique Targets	Largest Design
Malonyl-CoA	89	24
Myristoyl-CoA (14:0)	132	32
Myristoleoyl-CoA (14:1)	120	33
Palmitoyl-CoA (16:0)	144	25
Palmitoleoyl-CoA (16:1)	131	28
Stearoyl-CoA (18:0)	103	28
Oleoyl-CoA (18:1)	96	21

Number of targets produced by running Redirector for several fatty acid related products. **“**Unique targets” is the total number of unique enzyme targets found by Redirector using neighborhood sizes 1 to 6 and sensitivity and flat coefficient libraries. “Largest design” represents the largest group of targets found by redirector with a single neighborhood size and one coefficient library.

### Fatty Acid Production Network

**Figure 3:**
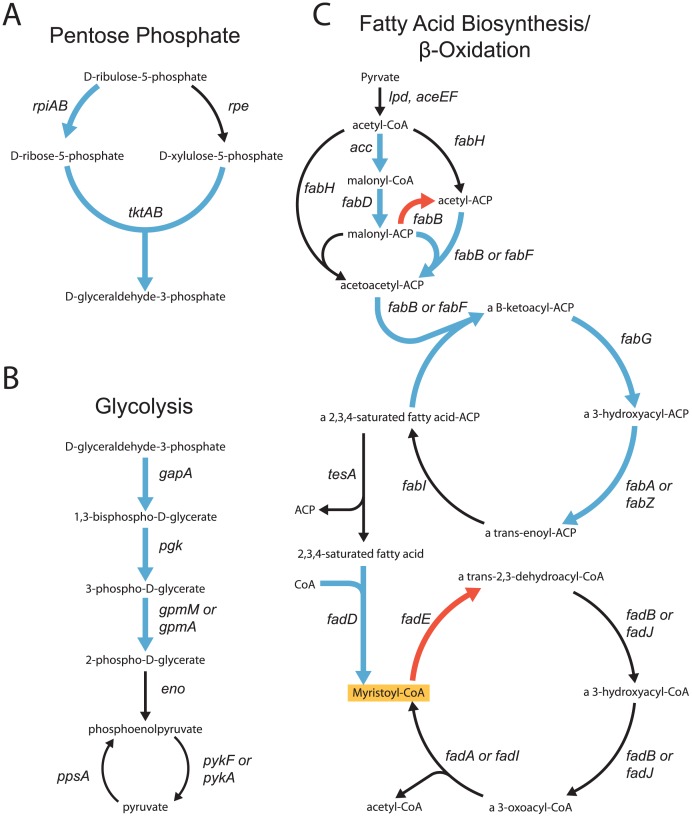
Pathways affected by an optimization of myristoyl-CoA. This optimization was run at neighborhood size 6 with flat coefficient library. Blue arrows indicate increased enzymes while the red arrows are decreased. The orange box indicates the production objective. A. Pentose phosphate pathway B. Glycolysis C. Fatty acid biosynthesis and β-oxidation.

To show an illustrative example of a Redirector design, we optimize for the production of myristoyl-CoA, and chose the design from the optimization results found after 15 iterations using the largest search size *k* = 6. This design achieved one of the highest levels of the progressive growth parameter and has 20 simultaneously active targets. Most of the enzyme targets found fall into one of 4 pathways: pentose phosphate, glycolysis, fatty acid degradation or fatty acid biosynthesis. The network in Figure 3 focuses on these pathways showing 15 of the 20 active targets (full list of targets in Table 2). We track targets by gene identifiers, which produce the enzyme being targeted, because of the intuitive concise naming of gene identifiers. For this analysis maximum glucose uptake allowed was set to 8.0 µmol/gDW/h resulting in the production of myristoyl-CoA of 1.54 µmol/gDW/h, reaching 80% of theoretical maximum yield, while maintaining 20% biomass yield. Additional information about the experimental confirmation of metabolic alterations is given in Supporting Information Table S7 and uptake and production fluxes for this design are shown in Supporting Information Table S8.

**Table 2:** Redirection coefficient values and effects.

Enzyme Name	Flat	Power Series	Total Flux Change	Flux Change Count
Biomass	155.25	186.41	−0.59	(−): 1, no change: 0, (+): 0
fabF or fabB	1	1.5	9.87	(−): 3, no change: 2, (+): 7
fabZ or fabA	1	0.5	9.39	(−): 3, no change: 2, (+): 7
fabG	1	1.5	9.34	(−): 3, no change: 2, (+): 7
fabD and acpP	1	1.5	9.34	(−): 0, no change: 0, (+): 1
accABCD	1	0.5	9.34	(−): 0, no change: 0, (+): 1
gpmA or gpmI or gpmB	1	0.5	2.11	(−): 0, no change: 0, (+): 1
aspC	−1	−0.5	1.72	(−): 0, no change: 0, (+): 1
fabK or fadD	1	0.5	1.54	(−): 0, no change: 9, (+): 1
gapA	1	0.5	1.09	(−): 0, no change: 0, (+): 1
pgk	1	0.5	1.09	(−): 0, no change: 0, (+): 1
tktB or tktA	1	0.5	1.09	(−): 0, no change: 0, (+): 2
ppk	1	1.5	0.30	(−): 0, no change: 1, (+): 1
rpiA or rpiB	−1	−1.5	0.12	(−): 0, no change: 0, (+): 1
fadE	−1	−1	0.00	(−): 0, no change: 8, (+): 0
acs	−1	−0.5	0.00	(−): 0, no change: 1, (+): 0
idi	−1	−0.5	−0.001	(−): 1, no change: 0, (+): 0
fabB	−1	−0.5	−0.27	(−): 3, no change: 1, (+): 0
folD	−1	−0.5	−1.20	(−): 2, no change: 0, (+): 0
gdhA	−1	−0.5	−5.04	(−): 1, no change: 0, (+): 0
acnB or acnA	−1	−1.5	−8.72	(−): 2, no change: 0, (+): 0

Values of flat and power series redirection coefficients for selected target enzymes found in the optimization for myristoyl-CoA, neighborhood size 6 at iteration 15. Total flux change represents the summation of all flux changes to the reactions that the enzyme controls for the flat redirection coefficients. Flux change count gives an overview of how the fluxes, associated with each enzyme, change as a result of this design. Flux change is calculated by comparing the value of the flux at current optimal system objective to those found during optimal growth. We indicate the number of reactions with flux levels that decrease (−), stay the same (no change), or increase (+).

In Figure 3 A and B, the pentose phosphate and glycolysis pathways bring in material from the carbon source, glucose, and process it for use in core metabolism, amino acids, fatty acid synthesis and other pathways. The targets in the pentose phosphate pathway (up-regulation of *rpiAB* and *tktAB)* act to produce NADPH, which is needed for fatty acid production, as well as drive flux towards D-glyceraldehyde-3-phosphate. The overproduced D-glyceraldehyde-3-phosphate from the pentose phosphate pathway leads directly into glycolysis, indicating a direct link between the engineering changes in the two pathways. In glycolysis (Figure 3B) the up-regulation targets *gapA*, *pgk*, and (*gpmM or gpmA*) drive carbon flux towards pyruvate and acetyl-CoA. Up-regulation of both *pgk* and *gpmAM* is an example of the Redirector framework's ability to discover multiple targets in a non-branching, linear pathway in the iAF1260 model. Up-regulating *pgk* increases production of 1,3-bisphospho-D-glycerate, which is only used in the reaction catalyzed by *gpmAM*. As mentioned previously finding multiple simultaneous targets in a linear pathway is a proven engineering strategy and would not be found if metabolic alterations are modeled using flux boundaries.

Figure 3C shows the fatty acid initiation, biosynthesis and β-oxidation pathways from pyruvate, up-regulated through glycolysis, to myristoyl-CoA. This design increases flux into the initiation of fatty acid biosynthesis through the up-regulation of *accABCD*, *fabD* and (*fabB* or *fabF)* pulling flux from acetyl-CoA and towards fatty acid production. We also find intuitively rational targets for fatty acid biosynthesis including up-regulation of (*fabB* or *fabF*), *fabG*, and (*fabA* or *fabZ)*, and discover proven fatty acid production targets, such as up-regulation of *fadD* as well as the knockout of *fadE*. While the enzyme group (*fabB* or *fabF*) is increased, *fabB* alone is decreased in this design. This seeming contradiction is a function of working with enzyme targets since (*fabB* or *fabF*) is recognized as one enzyme group that controls one set of reactions while *fabB* alone is recognized as a separate enzyme in the iAF1260 *E. coli* model. A simple way to implement this would be to up-regulate *fabF* while down-regulating *fabB*. In addition to these targets in closely linked pathways, we discover two targets, *folD* (formylTHF biosynthesis) and *idi* (isoprenoid biosynthesis) (Table 2), that are down-regulated in distant competing pathways.

The metabolic engineering targets shown in Figure 3 are examined in Table 2 to demonstrate the importance of the objective control method for modeling metabolic alterations in a manner which incentivizes, but does not force, flux changes. This approach is shown to be especially important when enzymes effect multiple chemical reactions. Up-regulation of the fatty acid biosynthesis enzymes (*fabA* or *fabZ*), *fabG* and (*fabB* or *fabF*) works to drive flux though the whole of fatty acid biosynthesis, 12 reactions for each enzyme. In the final optimization design each of these enzymes has been increased to one level, incentivizing all the associated reactions. We compare the final set of flux values resulting from this Redirector optimization to fluxes resulting from the optimization for biomass. This analysis indicates that only the fluxes through 6 reactions upstream of myristoyl-ACP increase, while the fluxes through 3 reactions for biosynthesis of larger unsaturated fatty acids do not change, and the fluxes through 3 reactions producing unsaturated fatty acids actually decrease.

Fluxes through the longer chain fatty acids, bigger than C14:0, do not increase, as we have added an export reaction for myristoyl-CoA into the model, such that myristoyl-CoA can be exported when it is overproduced. This selectivity for the carbon chain size of fatty acids and biofuel product export is biologically relevant. It has been shown that, with up-regulation of *tesA*, fatty acids are redirected from biomass and converted to free fatty acids [51]. The thioesterase protein, TesA, has chain length specificity for myristoyl-ACP, such that it preferentially acts to remove the ACP group from myristoyl-ACP over longer chain length substrates [5]. The *fadD* enzyme then catalyzes reactions leading to the addition of the Coenzyme A to the C14:0 free fatty acid. Both *fadD* and *tesA* are up-regulation targets found in separate optimizations and, along with knockouts interrupting the β-oxidation of fatty acids (ΔfadE), ensure that myristoyl-CoA is overproduced while no product with chain length larger than myristoyl-CoA will be produced in excess. This result matches with experimental analysis showing that the combination of these metabolic engineering targets result in the preferred production of C14 fatty alcohol [5].

Table 2 illustrates the complex result, from the described myristoyl-CoA redirector design, of incentivizing an enzyme that mediates several reactions. A number of the fluxes through these reactions, catalyzed by the same target enzyme, change by different amounts and in different directions. For example, of the 10 reactions mediated by *fadD* only 1 shows up-regulation. An optimization for myristoyl-CoA using a method that directly changes flux bounds, in the same manner, for every reaction catalyzed by an enzyme would prove difficult. For example, increasing the flux bounds for every reaction mediated by any of the fatty acid biosynthesis enzymes (fabAZ, fabBF or fabG), would cause some of these reactions to draw flux away from the intended product.

### Dependency Network Mapping

**Figure 4:**
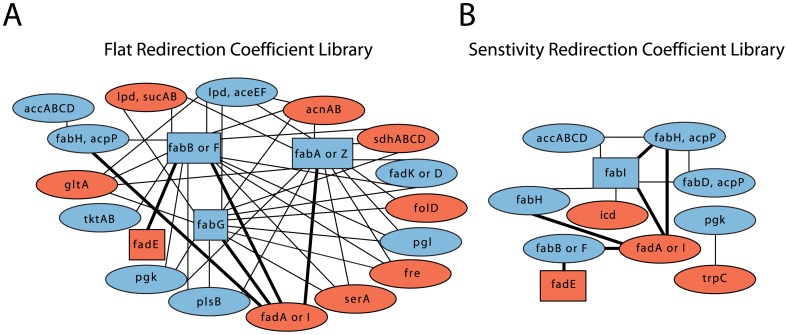
Enzyme group dependencies, of those enzymes that function alone and in pairs, for the optimization of myristoyl-CoA. Boxes indicate those enzymes that can work alone while the ovals are those enzymes that require one other enzyme to increase myristoyl-CoA production. Those enzymes in blue are increased while those in red are decreased. Darkened lines indicate the dependency groups which produce at least 90% of maximum output of myristoyl-CoA. A. Enzyme group dependencies for optimization using a flat redirection coefficient library. B. Enzyme group dependencies using a sensitivity redirection coefficient library.

The order in which genetic manipulations should be targeted is important information since the efficacy of some genetic alterations can depend on other genetic changes being made beforehand. Genetic manipulation is often carried out serially with a selection, and if there is an order of efficacy for these targets it is important to understand that order for the selection to work. This effect is illustrated when trying to produce fatty alcohols in *E. coli*, where knocking out *fadE* has no effect on fatty alcohol production unless *fadD* and *tesA′* have been up-regulated first [5]. Therefore, to develop a better relational understanding of the metabolic engineering targets discovered by redirector, which span a number of pathways with complex interrelationships, we use combinatorial analysis to find target sets that form the basis of larger designs. We constructed a network dependency map analyzing 132 targets from all of the separate designs that result in production of myristoyl-CoA. Figure 4 shows the dependency targets using undirected graphs for single enzymes as well as pairs. The links between singletons and other targets indicate that the production flux of the singleton target is improved by the addition of the secondary target. A small subset of all discovered targets are part of these single or double core target combinations, many more are involved in the triple sets or are only discovered to support production when the growth parameter is increased.

Figure 4A is the undirected graph of targets found searching neighborhood sizes 1 to 6 for 15 iterations using the flat redirection coefficient library. The flat coefficient library draws equally from all possible enzyme targets. Hence, we find a larger pool of targets than when using the sensitivity redirection coefficient library, Figure 4B. These 20 dependency targets focus on similar pathways, as discussed for Figure 3. There are enzyme targets enhancing the fatty acid biosynthesis pathway through the up-regulation of *accABCD*, (*fabA* or *fabZ*), (*fabB* or *fabF*), (*fabD, acpP*), (*fadK* or *fadD*), *fabG* and (*fabH*, *acpP*), as well as decreasing fatty acid degradation by reducing *fadE* and (*fadA* or *fadI*). The pentose phosphate pathway is enhanced through the up-regulation of *tktAB*, *pgl*, pgk and (*lpd*, *aceEF*) which drives flux towards acetyl-CoA. The TCA cycle is generally repressed via the reduction of *acnAB*, *icd*, (*lpd*, *sucAB*), (*sdhABCD*) and *gltA*. The down-regulation of *fre* results in the presence of more NADH and NADPH, which are used in the fatty acid biosynthesis cycle. The down-regulation of *serA* acts to halt animo-acid pathways from drawing flux from the pentose phosphate pathway, in particular 3-phospho-D-glycerate which is the direct product of the *pgk* enzyme.

The sensitivity redirection coefficient library dependency targets, while sparser, also center on fatty acid biosynthesis and degradation targets with the exception of *icd*, *pgk*, and *trpC*. The *trpC* enzyme is part of the L-tryptophan synthesis pathway and *icd* is part of the TCA cycle, both of which direct resources away from fatty acid synthesis. The combination of *trpC* and *pgk* is not found by the flat coefficients and may be a result of a finely tuned balance of forces from the sensitivity redirection coefficient library. Figure 4B shows a higher percentage of pairs with 90% production or higher (the darker lines). This is due to the fact that the sensitivity coefficient library is calculated to have more direct impact on the production of fatty acids.

In order to give a more complete picture of the dependency sets we present Table 3, which shows the dependency analysis from optimizations including enzyme sets up to size 3. No dependency sets were found which needed 4 or more targets as we don't employ a changing progressive growth parameter (γ) in the dependency network mapping. The triplicate sets are those that see improvement over any of the pairs or single targets that compose them. The trends of the dependency size 2 sets can be seen to continue in size 3, with fatty acid synthesis and degradation being the key players as well as the continued inclusion of the *gltA*, (*lpd*,*aceEF*) and *acnAB* group and the *pgk* and *trpC* group. The *pgk* and *trpC* group also combines with two new targets *pssA* and *psd*. Both are down-regulated and result in the repression of the lipid biosynthesis pathways which reduces the use of fatty acids for lipids.

**Table 3:** Dependency analysis results for targets from optimizations up to neighborhood size three of myristoyl-CoA with flat and sensitivity coefficient library.

Dependency Set	Dependency Size	Sensitivity/Flat	Production
fadA or fadI, fabH	2	Sensitivity	1.54
fadA or fadI, fabB or fabF	2	Both	1.54
fabB or fabF, fadE	2	Both	1.54
acpP and fabH, fadA or fadI	2	Both	1.54
fabI, fadE, fabB	3	Sensitivity	1.54
fabH, fadE, fabB	3	Sensitivity	1.54
fabA, fabI, fadE	3	Sensitivity	1.54
fabA, fabH, fadE	3	Sensitivity	1.54
fabA, fadA or fadI, fabI	3	Sensitivity	1.54
acpP and fabH, fadE, fabB	3	Sensitivity	1.54
acpP and fabH, fabA, fadE	3	Sensitivity	1.54
fadE	1	Both	1.54
fadA or fadI, fabI	2	Sensitivity	1.51
fabG, fadA or fadI	2	Flat	1.51
fadA or fadI, fabA or fabZ	2	Flat	1.51
trpC, pssA, pgk	3	Sensitivity	1.15
trpC, pgk, psd	3	Sensitivity	1.15
trpC, pgk	2	Sensitivity	1.13
gltA, aceEF and lpd, pgk	3	Flat	1.02
aceEF and lpd, pgk, acnAB	3	Flat	1.02

Dependency Set represents enzyme groups, separated by commas, that work together to improve myristoyl-CoA production. Dependency Size is the number of enzymes in the set that achieve the production level. The “Sensitivity/Flat” column represents the coefficient library which produced the dependency set. The Production column is the level of myristoyl-CoA resulting from the associated dependency set in µmol/gDW/h. These results represent a fraction of the total results, those which produce a relative production level at or above 1.02 µmol/gDW/h.

### Contrasting Redirector with Boundary Analysis

We further illustrate the importance of the Redirector approach, using objective control, by contrasting it with the limitations of using flux boundaries to model metabolic alterations. Further details beyond those presented here are included in the Supporting Information Text S1 section Fatty Acid Production Using Flux Constraints and in Supporting Information Tables S5 and S6. We performed a small scale analysis for the production of myristoyl-CoA using flux bounds to model metabolic alterations. In this analysis we use experimentally proven targets and likely targets in the fatty acid biosynthesis and degradation pathways. Flux bounds, minimum and maximum possible flux values for each reaction, were found while maintaining a certain percentage of flux through optimal biomass (i.e. 80% optimal biomass flux), and the same was done while maintaining a certain percentage of optimal myristoyl-CoA production. These boundaries on each reaction were compared between the biomass optimal and production optimal cases. Higher, non-overlapping bounds for the metabolite production case when compared to the biomass production case indicate that a reaction is a target for up-regulation. Lower, non-overlapping bounds for production would mean that a reaction is a target for down-regulation.

This method proved to be problematic in two ways (Table S5). First, when comparing flux boundaries while maintaining 80% or lower of either metabolite or biomass production, no viable targets for metabolic alteration were found. Second, when using flux boundaries to categorize reactions catalyzed by the same fatty acid biosynthesis enzyme, this analysis indicated that short chain reactions must be up-regulated, medium chain reactions were not valid targets for alteration and long chain reactions must be down-regulated. This makes implementing these bounds as a group for any of these enzymes hard to interpret has having any biological meaning. Finally, using these sets of boundaries to model metabolic alterations, we found that only the designs consisting of ΔfadE combined with one of these unrealistic constraints (fabAZ or fabBF or fabG) resulted in any production. Furthermore, no improvement in production was found by adding proven targets (accABCD, fadD or tesA), (Table S6). This meant that none of these experimentally proven targets could be included as meaningful metabolic alterations in designs found using this approach. For the above reasons it has been shown that current methods, such as OptForce, which model metabolic alterations using flux bounds would not succeed in optimizing products from complex pathways such as fatty acids without careful changes to the model, excluding target possibilities and picking certain targets in a non-automated fashion [52].

## Discussion

The Redirector framework provides a new capability in modeling metabolic alterations, using the FBA objective. This capability is harnessed to develop designs incorporating many metabolic alterations, which work in concert to drive flux in new directions and result in high production cellular metabolic factories. The objective control approach provides a more biologically relevant model of metabolic alterations, avoiding the unrealistically unlimited impact of changing flux boundaries. The Redirector framework is able to successfully develop designs for pathways where multiple chemical reactions are catalyzed by single enzymes, such as those that have elongation cycles, or complex branching or alternative pathways such as fatty acid metabolism. These designs find experimentally proven combinations of engineering targets along with novel targets in intuitive as well as distant pathways. Analyzing orthogonal and overlapping designs discovered by the framework, target dependency network mapping elucidates the relative importance and relationships of metabolic targets, in order to guide metabolic engineering. All together these methods form an effective, flexible and widely applicable framework for developing metabolic engineering designs for high production strains.

To demonstrate its capacity to optimize challenging pathways, we have applied Redirector to the production of myristoyl-CoA, examining the highest growth parameter design as well as the dependency analysis of multiple designs. The single high growth parameter design rediscovers experimentally proven combinations of targets including up-regulation of *acc*, *fabA* and *fabD* as well as reduction of *fadE*. Acetyl-CoA carboxylase (*acc*) catalyzes the first committed step toward fatty acid biosynthesis. Up-regulation of *acc* leads to an increase in overall fatty acids [51] and is part of most fatty acid and fatty acid based biofuel production designs [5], [6]. Up-regulation of *fabA*, 3-hydroxydecanoyl-[acp] dehydrase, has been shown to increase the level of fatty acid metabolites present in the cell [53]. The combination of up-regulation of *fadD* while knocking out *fadE*, has been shown to result in biofuel production from acyl-CoA precursors [5]. Detail on the experimentally proven targets found by Redirector can be found in Table S8 in the Supporting Information.

Looking at the single myristoyl-CoA design in Table 2, and the dependency analysis of multiple myristoyl-CoA designs (Table S2), we observe targets in intuitive, and more distant, supporting pathways. A number of these targets have been experimentally shown to enhance malonyl-CoA (a precursor to myristoyl-CoA) production, including reduction of *fumABC*, *acs*, *sucAB*, *acnAB*, glyA and *sdhABCD* as well as increasing *aceEF*, and *pgk* [48], [54]. Table 2 also shows targets which support production less directly, as it redirects flux down the pentose phosphate pathway to balance the redox needs of enhanced fatty acid biosynthesis. Redirector also finds reduction targets, such as *idi* (isoprenoid biosynthesis) and *folD* (formylTHF biosynthesis), to reduce competition for carbon. Previous computational production designs have used *folD* knockouts to prevent flux away from 3-p-Glycerate towards formate [55]. Similarly, enhancing expression of *idi* has been shown to increase production of isoprenoids [9], which would compete with fatty acid production. We can conclude that the Redirector approach is able to develop effective designs in challenging pathways, which bring together diverse and complex connections in the metabolic network in order to drive flux towards production.

Using objective control, Redirector pushes fluxes in new directions finding ever higher impact metabolic engineering designs. Alternatively, OptForce, a method with similar goals as Redirector, iteratively constrains the system to find minimal sets of reactions that force more flux into the desired product. We have shown that using flux bounds based models of metabolic alterations proves challenging for fatty acid production for three main reasons. First, if flux bounds are not sufficiently strict, no viable constraint sets will be found. Second, the flux bounds discovered can lead to unrealistic constraints when mapping reactions to causal enzymes. Third, limits do not work additively which restricts the number of targets that can work together in any one design, causing experimentally proven targets for fatty acid production to be missed. To further compare these methods, we applied the Redirector framework to the production of malonyl-CoA, which has been optimized as a precursor for the production of the flavonoid naringenin using OptForce [48]. Redirector is able to discover most of the experimentally validated targets found by OptForce in a single design (Supporting information Table S1), including up-regulation of *acc, pgk*, (*aceEF* and *lpd*), and reductions of *acnAB*, *fumABC*, as well as reduction of *sucCD* in an alternate design path. The only experimentally tested OptForce target not found by Redirector was reduction of *mdh*. However, this target makes little improvement on its own and seems to hurt production when combined with any of these other targets experimentally. Redirector also finds experimentally validated targets not found using the OptForce approach, including the reduction of *glyA* (*g*lycine production) and fatty acid biosynthesis initiation (*fabBF*) as well as reduction of *sdhABCD* (TCA cycle) in the dependency network mapping (Supporting Information Table S2). In a strain over-expressing known malonyl-CoA production targets (*acc* and *bpl*), individual knockouts of *sdhABCD* or *glyA* result in an additional ∼200% or ∼300% (mg/L/OD) respective increase in naringenin production [54]. Reduction of fatty acid biosynthesis initiation, using cerulinen has been shown to enhance production of naringenin by ∼280% (mg/L) [56], [57]. We conclude that Redirector performs well for products near core metabolism while excelling in optimizing more distant products, those outside core metabolism, from pathways with complex enzyme/reaction relationships.

The breadth of malonyl-CoA production target combinations also allows us to further compare our dependency network mapping to experimental results for engineering target interdependencies, elucidating those targets which are required for others to be effective. Looking at the target dependency network mapping (Supporting Information Table S2), we observe the core targets for producing malonyl-CoA are the up-regulation of *accABCD* and down-regulation of fatty acid biosynthesis initiation (*fabB* or *fabF*, *fabD* or *fabK*, *fabH*). These targets exclusively make up the size 2 dependency sets and participate in most size 3 dependency sets. The importance of these two groups is quite intuitive as *accABCD* directly produces malonyl-CoA while fatty acid biosynthesis draws directly from malonyl-CoA. The primacy of these targets is confirmed in successful metabolic engineering strategies. Overexpression of acetyl-CoA carboxylase, or repression of fatty acid biosynthesis initiation have been sufficient to increase naringenin production 2 to 3 fold [56], [57]. These are the only targets found to increase production to this level individually. Other targets achieve similar levels of improvement only when combined with acetyl-CoA carboxylase overexpression [48], [54]. In the target dependency network mapping, we also observe that up-regulation of *accABCD* can be enhanced but not replaced by overexpression of targets upstream of acetyl-CoA. This interdependency is in direct agreement with experimental evidence. Comparing dependency network mapping to experiment demonstrates that the Redirector method discovers realistic, minimal sets of core targets and the manner in which further targets can build on this core.

Redirector makes it possible to model metabolic alterations in a manner more closely representing reality as changes to the catalytic landscape of the metabolic system, resulting in a new balance between synthetically created, and natural existing cellular drives. Redirector is able to represent the impact of metabolic alterations as redirection coefficients, which can be associated with each enzyme target and possible metabolic alteration. This model of balanced and interacting impacts is critical to discovering experimentally proven combinations of engineering targets. We have also shown that this objective control model of metabolic alterations is critical for enabling optimization of enzyme targets when enzymes catalyze multiple reactions. We observed when enhancing fatty acid biosynthesis, fluxes incentivized by the same enzyme had necessarily varying responses. These included reactions controlled by the same enzyme changing in opposite directions, as some increase, while other fluxes are limited by network topology or are reduced in response to changes in biomass production.

The Redirector method complements experimental techniques in strain design and development. In particular high throughput genetic manipulation techniques such as MAGE [9] can alter tens of targeted sites simultaneously in only a few rounds of recombination and would benefit from large sets of targets generated by Redirector. The Redirector frameworks can be leveraged to best inform which genes to target for metabolic engineering, the direction of the manipulation (up-regulation or down-regulation), the relative magnitude of the engineering instruction and the order in which they can be targeted. The level of up-regulation or down-regulation suggested by Redirector can be a guide to processes such as using MAGE to alter the RBS or promoter of a gene, skewing the sequences toward increased or decreased expression. The large number of targets found with progressive target discovery, when combined with advances in metabolic engineering and the consideration of natural regulatory mechanisms, give the metabolic engineer additional options to produce high production strains and increase the potential for greater strain stability. Looking forward, Redirector opens a number of promising avenues. The concept of balancing various forces is widely used in predictive modeling frameworks in other fields of study, and similarly various forms of our objective control method are broadly applicable in metabolic optimization design. Areas of obvious promise include optimization of microbial communities though balancing of objectives in different strains as they compete and exchange metabolites, balancing environmental and genetic changes, and developing a better model of natural cellular behavior as a balance of competing aspects of regulatory programing.

## Materials and Methods

### Objective Control

Here we develop a novel and more biologically relevant model for representing alterations to the metabolic system. Rather than modeling metabolic alterations as directly changing reaction flux values or boundaries, the Redirector framework uses changes in an objective function, in which both engineered enzyme targets and natural biological objectives are represented. Such an objective describes an organism that has to allocate its resources to achieve a compromise between its natural cellular programming and the alterations imposed on it by human engineering, aiming to generate a desired product. This combined objective of the system is represented as:
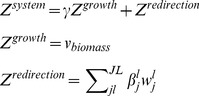
(1)


The system objective Z^system^ reflects the combination of the growth function Z^growth^ and the metabolic engineering changes modeled in the redirection function Z^redirecton^. In this paper the growth function is the standard FBA biomass production flux, v_biomass_. We refer to γ as the progressive growth parameter, and it is used to tune the relative contribution of the growth function in the system objective which becomes important in the progressive target discovery. J is the set of all metabolic reactions *j* in the system and L is the set of all metabolic changes l in the system.

The impact of metabolic engineering alterations is represented by fluxes included in the redirection function. Each flux *v_j_* in the redirection objective is weighted by one or more ‘redirection’ coefficients β_j_^l^. One of the design goals of Redirector is to represent engineering changes as modifications that cause resources to be diverted away from growth to desired end products. Yet, using metabolic engineering, the magnitude of diversion cannot be forced between specific bounds. Furthermore, the flux through any particular reaction also depends on the balance of fluxes throughout the metabolic network. Redirection coefficients support this design goal because they operate only as incentives to increase or decrease target fluxes rather than hard constraints. Redirection coefficients can be thought of as ‘impact factors’ for the engineered changes, as stronger metabolic alterations can be represented as larger redirection coefficients, which will generate greater contributions to the total redirection function, in turn providing stronger incentives for a metabolic network to direct flux through the corresponding reaction.

To represent the effects of metabolic engineering changes of different magnitude and direction, each reaction *j* can be assigned multiple redirection coefficients β_j_^l^ each uniquely identified by l. Though each reaction can have multiple redirection coefficients, they are added together to form a single level that will be suggested as the metabolic alteration instruction for that reaction, as described below. Reaction fluxes are included in the redirection function through the use of the objective inclusion variable *w_j_^l^*. When reaction *j* and the associated metabolic change identifier *l* are included in the redirection function then *w_j_^l^* = *v_j_*, where *v_j_* is the flux of reaction *j*, otherwise *w_j_^l^* = 0. In the case that a reaction flux *j* is chosen as a target for metabolic engineering change *l*, β_j_^l^ *v_j_* is effectively included in the redirection function. The set of all possible redirection coefficients β_j_^l^ allowed for a particular optimization is referred to as the “redirection coefficient library”.

Using this formulation Redirector can also allow for the selection of multiple redirection coefficients for the same reaction. The final contribution of this reaction to the redirection function is then equal to the sum of the associated redirection coefficients. The sum of the associated coefficients is then considered the one singular suggested genetic manipulation for that reaction in the final target solution set with a relative strength equal to the summed value. Using multiple coefficients for the same reactions allows tuning of the level of up-regulation or down-regulation suggested for one reaction during the optimization.

This formulation of the system objective allows us to create and control which reactions can be included in the redirection function, as well as their possible contribution to the redirection function. Specifically these factors are determined by choosing the contents of the redirection coefficient library. The number of reactions considered for inclusion in the redirection function can be narrowed by limiting the reactions that get redirection coefficients or broadened by allowing multiple redirection coefficients for each flux to be included in the redirection function. The number of coefficients for each reaction flux is determined using a coefficient tuning variable (s) described in the Supporting Information Text S1 section Bilevel Optimization Problem. The objective control method for representing metabolic alterations forms the basis for selecting optimal sets of enzyme targets using a bilevel optimization and is described in the Supporting Information Text S1.

### Redirection Coefficient Library

The Redirector framework selects the optimal enzymes and metabolic changes and includes the related reaction fluxes in the redirection function by choosing from a list of possible redirector coefficients as set out in the redirection coefficient library. The inclusion of a positive redirection coefficient for a flux in the redirection function incentivizes increase of that flux (this would also penalize a decrease in flux) and a negative redirection coefficient has the opposite effect. Discovery of the optimal magnitude of redirection coefficients informs the relative strength of metabolic engineering changes. The optimal set of enzyme targets is determined as much by the reactions affected, as it is by the possible redirection coefficients in the redirection coefficient library. Further details of these redirection coefficient libraries can be found in the Supporting Information Text S1 section Redirection Coefficient Library.

### Progressive Target Discovery

We extend the bilevel implementation of objective control to find progressively higher impact sets of interacting targets. This is achieved by harnessing the competition between the two parts of the system objective, in which the growth function directs resources to the biomass, while the redirection function directs resources to the metabolite production. Once an optimal set of metabolite production targets has been discovered and the production objective is at its optimal possible level, no more targets will need to be discovered. However, increasing the relative strength of the growth function will necessitate selecting more enzyme targets to once again achieve high metabolite production. To this end, we expand the iterative local search method to include the adjustment of the progressive growth parameter (γ). At each iteration, γ is increased to a value where more targets must be selected to increase the strength of the redirection function and satisfy the production objective. This allows the Redirector framework to build upon the set of enzyme targets at each iteration. The combined effect of these targets results in ever increasing set of incentives to drive flux towards the production objective until the maximum potential redirection function is reached.
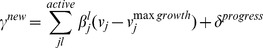
(2)


To drive the discovery of new targets we seek to increase the value of the progressive growth parameter (γ) such that the growth term will dominate the system objective. This is achieved by finding a new value of the progressive growth parameter, γ^new^, that will result in an effective growth function value which is at least slightly larger than the contribution of the current redirection function to the system objective. The first term in the equation is the current strength of the redirection function. This term is calculated as the included redirection coefficients β_j_^l^ multiplied by the difference in current flux v_j_ and the flux when growth is maximized v_j_^max growth^, for each included reaction j. The variable δ^progress^ is a small number used to insure γ^new^ is slightly larger than the current strength of the redirection function. This new progressive growth parameter will increase the effective strength of the growth function in the system objective such that overcoming it requires the inclusion of new reaction fluxes in the redirection function as a result of new enzyme targets being selected. The logical flow of the Redirector iterative search local algorithm, which incorporates the progressive growth parameter, is detailed in Figure 1 and the Supporting Information Text S1. We show that the algorithm is robust against changes in the δ^progress^ and γ parameters in Supporting Information Table S6.

### Dependency Network Mapping

Many enzyme targets generated during the progressive target discovery depend on the inclusion of other core targets before they can contribute to an increase in the production objective. To determine the order in which targets should be engineered as well as their interdependency we develop a dependency network mapping method.

To carry out the process of dependency network mapping all targets for one production objective from separate neighborhood sizes and redirection coefficient libraries are pooled in any relevant combination. Then all subsets of this target pool up to size N are searched in separate optimizations, by forming the relevant system objective and performing a single-level FBA with this objective. The resulting flux states are checked to discover if the target combinations result in at least 20 percent of the maximum possible production of the metabolite of interest and, importantly, if they result in higher production than their component target sets. Through this analysis we discover which targets work as singles, doubles etc. In this way, dependency network mapping shows which enzymes form the core of metabolic production designs. Currently we focus our dependency network mapping on the discovery of dependency target sets needed to produce the metabolic product when γ = 0.02; thus, we only require that each engineering design needs to overcome a very weak growth function. As a result, the large sets of simultaneous targets found in progressive growth driven target discovery, which require larger values of γ, are not rediscovered.

### Software and Hardware

The Redirector framework is built using free, and whenever possible, open software in a flexible lightweight solution. The core is built with Python and currently uses the GLPK and SCIP solvers. LP optimizations were largely carried out in GLPK because of the ability to directly access GLPK functions from Python while MILP optimizations are carried out by SCIP for faster solving speed. Computation was carried out on the Broad Institute computational cluster. The Redirector Package including operational software code and metabolic network model files used for this publication are available at https://github.com//bionomicron/Redirector.git.

## Supporting Information

Figure S1 **Myristoyl-CoA optimization search time.** Search time in seconds vs. the iteration number for the optimization of myristoyl-CoA. The color of the lines indicates associated neighborhood size. A. Search time vs. iteration for myristoyl-CoA using the flat adjustment library. B. Search time vs. iteration for myristoyl-CoA using the sensitivity adjustment library.
(EPS)

Figure S2 **Fatty acid concentration in strains with metabolic alterations.** Relative fatty acid concentration, as measured with the Roche Free fatty acids, Half-micro test, in four different strains of *E. coli* with varying levels of anhydrotetracycline (ATc) induction. The blue bar represents EcfC1, a λ-red recombination strain, the green bar represents EcfC1 with *fadE* gene knocked out, the red bars represent EcfC1 with the *fadE* knockout and *tesA*′ and *fadD* overexpressed with ATc induction, and the dark brown bars represent EcfC1 with the *fadE* knockout as well as *tesA*′, *fadD* and *fadR* overexpressed with ATc induction. Gene expression was induced with 0, 3, 5, and 8 ng/ml ATc.
(EPS)

Table S1 **Malonyl-CoA optimization results.** List of targets from an optimization of malonyl-CoA at neighborhood size 3 and iteration 5. A. Optimization design results for the flat redirection coefficient. B. Optimization design results for the power series redirection coefficient. Total Flux Change represents the summation of all flux changes to the reactions that the enzyme controls. Flux Change Count gives an overview of how the fluxes, associated with each enzyme, change as a result of this design. Change is calculated by comparing the value of the flux at current optimal system objective to those found during optimal growth. We indicate the number of reactions with flux levels that decrease (−), stay the same (no change), or increase (+).
(DOCX)

Table S2 **Malonyl-CoA dependency network.** The double and triple metabolic alteration target dependency groups found for malonyl-CoA production are shown along with the predicted amount of production. There were no single enzymes that were found by the dependency analysis. Production indicates the amount of malonyl-CoA made by that dependency group.
(XLSX)

Table S3 **Variable summary.** This table gives an overview of the variables used in the Redirector method. The variable name, a basic description of the purpose of the variable, and how the value of the variable is determined are presented. The dependent variables of the bilevel optimization (*v*, *y*, *w*, *u*) are determined by solving the optimization problem.
(DOCX)

Table S4 **Progressive target discovery robustness.** This table illustrates the robustness of the Redirector method specifically the progressive target discovery to varying values of δ^progress^. Shown here are the targets discovered by the Redirector method for the production of myristoyl-CoA (C14:0-CoA), using a search size of 4 metabolic alterations (k = 4) during iteration 3 and 4 (i = 3,i = 4). The left most column indicates the gene id of the targets. Redirection coefficients for the selected targets and the sum of fluxes through the reactions associated with those gene ids are given in the other columns. The table shows the targets discovered and fluxes through the associated reactions are completely unchanged as δ^progress^ is varied over a ranged of four orders of magnitude.
(DOCX)

Table S5 **Boundary analysis.** This table shows a subsection of a boundary analysis we performed on the set of reactions catalyzed by enzymes which are either experimentally proven or likely targets to achieve production of myrstiol-CoA (C14:0). Boundary analysis was performed by minimizing and maximizing each flux, while restricting the value of the biomass or production flux to a percentage of their maximum value. Column A indicates the catalyzing enzyme while column B shows the catalyzed reaction, identified the reaction codes used in the iAF1260 model. The lower and upper bounds for each reaction, while maintaining 100% biomass production, are shown in columns C and D respectively. While columns E and F show the lower and upper bounds respectively for each reaction while maintaining 100% production of myristiol-CoA. Green highlighted rows indicate reactions that must be increased in order to achieve 100% production of myristoyl-CoA when compared to 100% biomass production, while orange highlighted rows indicate those that must decrease to do so. Un-highlighted rows have overlapping flux boundaries while maintaining optimal biomass or production flux. Additional boundary analysis was carried out for varying percentages of maximum biomass and myristoyl-CoA production shown in “supporting information table 1”.
(DOCX)

Table S6 **Myristoyl-CoA production with set boundary limits.** This table shows the production of myristoyl-CoA achieved by constraining all reactions associated with the set of enzymes listed in the first column within the boundaries found by boundary analysis. The percent constraint indicates the percentage of maximum production to which the network was constrained when doing the boundary analysis. For example 100% constraintis what was used in finding the boundaries shown in table S5 column E and F, since those boundaries were found while maintain 100% of maximum production flux. The production values are found by imposing the indicated combinations of boundaries on the iAF1260 model then optimized for minimization of the production of myristoyl-CoA (as done in the OptForce approach). All shown boundary combinations were tested in conjunction with a *fadE knockout*, without which no production was discovered.
(DOCX)

Table S7 **Experimentally proven overproduction targets from Redirector.** Redirector targets (target column) found for overproduction of different products (product column) are shown along with the base strain alterations needed to achieve the production experimentally and percent of the original production achieved. If no wild type strain is shown wild type production levels were too low to be a useful comparison (less than one percent of base line being used). Production levels are given as a percent of the original production of the strain being metabolically altered. Percentages were calculated by look at the ratio of total mg/L produced.
(DOCX)

Table S8 **Redirector example design update and production.** Uptake and export reactions fluxes comparing the flux distribution (optimal biomass), production flux distribution (optimal C14:0-CoA production) and the design used for Figure 1 found by the Redirector framework. To enhance numerical uptake numbers are kept near 10, all fluxes in this work can be projected maintain the same ratio to glucose and O2 uptake, if experimental conditions allow for greater uptake. For all optimizations a minimum of 20% of maximum biomass is maintained. Reactions are identifier names from the iAF1260 model are used. Units used in actual calculations are reduced by a factor of 10 to reduce numerical instability.
(DOCX)

Table S9 **Boundary analysis of known fatty acid production targets for various production levels.** Minimum and maximum possible flux values are shown for reactions catalyzed by known fatty acid production targets. Varying minimum and maximum values of reaction fluxes are given depending on required production level of either Biomass or C14:0-CoA. These requirements are implemented as lower boundaries placed on biomass and C14:0-CoA production, shown as a percent of their respective maximum possible flux values. The production required and the corresponding percentage are given for each column along with the term lower or upper, indicating the lower boundary or upper boundary on the possible flux space for the reaction in question.
(XLS)

Text S1 **Supplementary methods.** The text in this section provides additional information on the construction of the Redirector framework. First is a description of the construction of the redirection function. Second, the construction and usage of inclusion and exclusion variables is explained. It is then shown how these two elements are brought together into a Bilevel MILP formulation. Several methods for constructing redirection coefficient libraries are presented along with the cases in which each would be useful. Next, the iterative local search process used in the framework is presented, showing the progressive search method and how it is used. Finally, a list of variables used in the Redirector framework, and their importance, is given followed by some directions on best practices in using the Redirector approach. **Supplementary results.** This section includes a graph and discussion of Redirector optimization search times. A contrast of Redirector and flux boundary methods for production of fatty acids is examined. Finally, there is discussion of experimental validation of some of our targets.
(DOCX)
