# Supplementary material for: Redirector: Designing Cell Factories by Reconstructing the Metabolic Objective
Source: PLoS Comput Biol. 2013 Jan 17;9(1):e1002882. doi: 10.1371/journal.pcbi.1002882 (PMC3547792; doi:10.1371/journal.pcbi.1002882)
Supplement: Table S1 — Malonyl-CoA optimization results. List of targets from an optimization of malonyl-CoA at neighborhood size 3 and iteration 5. A. Optimization design results for the flat redirection coefficient. B. Optimization design results for the power series redirection coefficient. Total Flux Change represents the summation of all flux changes to the reactions that the enzyme controls. Flux Change Count gives an overview of how the fluxes, associated with each enzyme, change as a result of this design. Change is calculated by comparing the value of the flux at current optimal system objective to those found during optimal growth. We indicate the number of reactions with flux levels that decrease (−), stay the same (no change), or increase (+). (DOCX) [file pcbi.1002882.s003.docx]

**A)**

| **Enzyme Groups** | **Flat** | **Total Flux Change** | **Flux Count** |
| --- | --- | --- | --- |
| Biomass | 70.94733662 | -0.5894 | (-): 1, no change: 0, (+): 0 |
| accABCD | 1 | 14.1948 | (-): 0, no change: 0, (+): 1 |
| aceEF and lpd | 1 | 5.6507 | (-): 0, no change: 0, (+): 1 |
| gpmI or gpmB or gpmA | 1 | 3.8444 | (-): 0, no change: 0, (+): 1 |
| pflAB | 1 | 1.8451 | (-): 0, no change: 0, (+): 1 |
| trpA and trpB | 1 | 1.5510 | (-): 1, no change: 1, (+): 1 |
| thrB | -1 | 1.5084 | (-): 0, no change: 0, (+): 1 |
| Acs | -1 | 0.0000 | (-): 0, no change: 1, (+): 0 |
| nrdEF and grxA | 1 | -0.0627 | (-): 4, no change: 0, (+): 0 |
| fabD and acpP | -1 | -1.4221 | (-): 1, no change: 0, (+): 0 |
| glyA | -1 | -2.4943 | (-): 1, no change: 0, (+): 0 |
| Pgk | -1 | -2.5803 | (-): 1, no change: 0, (+): 0 |
| fumC or fumA or fumB | -1 | -4.3411 | (-): 1, no change: 0, (+): 0 |
| fold | -1 | -4.8562 | (-): 2, no change: 0, (+): 0 |
| acnB or acnA | -1 | -8.7229 | (-): 2, no change: 0, (+): 0 |

**B)**

| **Enzyme Name** | **Power** | **Total Flux Change** | **Flux Change Count** |
| --- | --- | --- | --- |
| Biomass | 113.0909 | -0.5894 | (-): 1, no change: 0, (+): 0 |
| accABCD | 1.75 | 14.1951 | (-): 0, no change: 0, (+): 1 |
| cynT or can | 0.25 | 13.9318 | (-): 0, no change: 0, (+): 1 |
| gpmI or gpmB or gpmA | 1 | 3.8444 | (-): 0, no change: 0, (+): 1 |
| Ppk | 0.5 | 1.8372 | (-): 0, no change: 1, (+): 1 |
| trpA and trpB | 0.5 | 1.5511 | (-): 1, no change: 1, (+): 1 |
| ybcF or yahI or yqeA | 0.5 | -0.3693 | (-): 1, no change: 0, (+): 0 |
| metL or thrA | -1 | -1.4178 | (-): 1, no change: 0, (+): 0 |
| acpP and fabD | -1.5 | -1.4222 | (-): 1, no change: 0, (+): 0 |
| tnaA | -0.125 | -1.6181 | (-): 1, no change: 0, (+): 0 |
| purN | -0.5 | -2.1090 | (-): 1, no change: 0, (+): 0 |
| glyA | -1.5 | -2.4944 | (-): 1, no change: 0, (+): 0 |
| sucC and sucD | -0.125 | -3.4183 | (-): 1, no change: 0, (+): 0 |
| gltA | -1 | -4.3615 | (-): 1, no change: 0, (+): 0 |
| fold | -0.5 | -4.8564 | (-): 2, no change: 0, (+): 0 |
| acnB or acnA | -1 | -8.7229 | (-): 2, no change: 0, (+): 0 |
| atpABCDEGHI | -0.75 | -24.5181 | (-): 1, no change: 0, (+): 0 |
