# Supplementary material for: Redirector: Designing Cell Factories by Reconstructing the Metabolic Objective
Source: PLoS Comput Biol. 2013 Jan 17;9(1):e1002882. doi: 10.1371/journal.pcbi.1002882 (PMC3547792; doi:10.1371/journal.pcbi.1002882)
Supplement: Table S3 — Variable summary. This table gives an overview of the variables used in the Redirector method. The variable name, a basic description of the purpose of the variable, and how the value of the variable is determined are presented. The dependent variables of the bilevel optimization (v, y, w, u) are determined by solving the optimization problem. (DOCX) [file pcbi.1002882.s005.docx]

| Variable | Function | Method of value determination |
| --- | --- | --- |
| *v* | Steady state flux | Dependent variable in LP |
| *y* | Binary control variable | Dependent variable in LP |
| *w* | Inclusion variable | Dependent variable in LP |
| *u* | Exclusion variable | Dependent variable in LP |
| $\beta$ | Redirection coefficient | Using flat, sensitivity or power series library construction method |
| *i* | Iteration number | Indicates the number of iterations of Redirector optimizations that have occurred. |
| *k* | Local neighborhood search size | Chosen to meet computational power. |
| *s* | Coefficient tuning variable | Selected to fit computational power. Usually only used with power series search. |
| $\gamma$ | Progressive growth parameter | Incremented to value to drive new target selection |
| δ^progress^ | Small term to insure $\gamma^{new}$ is larger than the current value of the redirector function | Chosen to be two orders of magnitude smaller than the initial gamma value it is incrementing. |
