# Supplementary material for: Redirector: Designing Cell Factories by Reconstructing the Metabolic Objective
Source: PLoS Comput Biol. 2013 Jan 17;9(1):e1002882. doi: 10.1371/journal.pcbi.1002882 (PMC3547792; doi:10.1371/journal.pcbi.1002882)
Supplement: Table S4 — Progressive target discovery robustness. This table illustrates the robustness of the Redirector method specifically the progressive target discovery to varying values of δprogress. Shown here are the targets discovered by the Redirector method for the production of myristoyl-CoA (C14:0-CoA), using a search size of 4 metabolic alterations (k = 4) during iteration 3 and 4 (i = 3,i = 4). The left most column indicates the gene id of the targets. Redirection coefficients for the selected targets and the sum of fluxes through the reactions associated with those gene ids are given in the other columns. The table shows the targets discovered and fluxes through the associated reactions are completely unchanged as δprogress is varied over a ranged of four orders of magnitude. (DOCX) [file pcbi.1002882.s006.docx]

| Test K4 I3 | δ=0.1 | Flux | δ=0.01 | Flux | δ=0.001 | Flux | δ=0.0001 | Flux |
| --- | --- | --- | --- | --- | --- | --- | --- | --- |
| accABCD | -1.00 | 8.94 | -1.00 | 8.94 | -1.00 | 8.94 | -1.00 | 8.94 |
| fadE | 1.00 | 0.00 | 1.00 | 0.00 | 1.00 | 0.00 | 1.00 | 0.00 |
| fabB | 1.00 | -0.27 | 1.00 | -0.27 | 1.00 | -0.27 | 1.00 | -0.27 |
| aceEF & lpd | -1.00 | 9.18 | -1.00 | 9.18 | -1.00 | 9.18 | -1.00 | 9.18 |
| Biomass | -25.08 | -0.59 | -23.64 | -0.59 | -23.41 | -0.59 | -23.41 | -0.59 |
| acnB or acnA | 1.00 | -2.68 | 1.00 | -2.68 | 1.00 | -2.68 | 1.00 | -2.68 |
| Test K4 I4 | δ=0.1 | Flux | δ=0.01 | Flux | δ=0.001 | Flux | δ=0.0001 | Flux |
| accABCD | -1.00 | 10.50 | -1.00 | 10.50 | -1.00 | 10.50 | -1.00 | 10.50 |
| fabF or fabB | -1.00 | 10.77 | -1.00 | 10.77 | -1.00 | 10.77 | -1.00 | 10.77 |
| fadE | 1.00 | 2.98 | 1.00 | 2.98 | 1.00 | 2.98 | 1.00 | 2.98 |
| fabB | 1.00 | -0.27 | 1.00 | -0.27 | 1.00 | -0.27 | 1.00 | -0.27 |
| aceEF & lpd | -1.00 | 10.83 | -1.00 | 10.83 | -1.00 | 10.83 | -1.00 | 10.83 |
| ydiD or fadD | 1.00 | 1.49 | 1.00 | 1.49 | 1.00 | 1.49 | 1.00 | 1.49 |
| Biomass | -39.31 | -0.59 | -36.09 | -0.59 | -35.74 | -0.59 | -35.74 | -0.59 |
| acnB or acnA | 1.00 | -1.02 | 1.00 | -1.02 | 1.00 | -1.02 | 1.00 | -1.02 |
