# Supplementary material for: Redirector: Designing Cell Factories by Reconstructing the Metabolic Objective
Source: PLoS Comput Biol. 2013 Jan 17;9(1):e1002882. doi: 10.1371/journal.pcbi.1002882 (PMC3547792; doi:10.1371/journal.pcbi.1002882)
Supplement: Table S5 — Boundary analysis. This table shows a subsection of a boundary analysis we performed on the set of reactions catalyzed by enzymes which are either experimentally proven or likely targets to achieve production of myrstiol-CoA (C14:0). Boundary analysis was performed by minimizing and maximizing each flux, while restricting the value of the biomass or production flux to a percentage of their maximum value. Column A indicates the catalyzing enzyme while column B shows the catalyzed reaction, identified the reaction codes used in the iAF1260 model. The lower and upper bounds for each reaction, while maintaining 100% biomass production, are shown in columns C and D respectively. While columns E and F show the lower and upper bounds respectively for each reaction while maintaining 100% production of myristiol-CoA. Green highlighted rows indicate reactions that must be increased in order to achieve 100% production of myristoyl-CoA when compared to 100% biomass production, while orange highlighted rows indicate those that must decrease to do so. Un-highlighted rows have overlapping flux boundaries while maintaining optimal biomass or production flux. Additional boundary analysis was carried out for varying percentages of maximum biomass and myristoyl-CoA production shown in “supporting information table 1”. (DOCX) [file pcbi.1002882.s007.docx]

| A | B | C | D | E | F |
| --- | --- | --- | --- | --- | --- |
| Gene / Enzyme | Row Names | 100%  Bio  Lower | 100%  Bio  Upper | 100%  C14:0-CoA  Lower | 100%  C14:0-CoA  Upper |
| accABCD | ACCOAC | 1.78 | 1.78 | 13.17 | 13.17 |
| acnAB | ACONTb | 4.52 | 4.52 | 0.00 | 0.00 |
| aceEF lpd | PDH | 6.93 | 6.93 | 15.36 | 15.36 |
| fabAZ | r3HAD40 | 0.26 | 0.26 | 2.19 | 2.19 |
| fabAZ | r3HAD60 | 0.26 | 0.26 | 2.19 | 2.19 |
| fabAZ | r3HAD80 | 0.26 | 0.26 | 2.19 | 2.19 |
| fabAZ | r3HAD100 | 0.26 | 0.26 | 2.19 | 2.19 |
| fabAZ | r3HAD120 | 0.15 | 0.15 | 0.00 | 2.19 |
| fabAZ | r3HAD121 | 0.11 | 0.11 | 0.00 | 2.19 |
| fabAZ | r3HAD140 | 0.09 | 0.09 | 0.00 | 2.19 |
| fabAZ | r3HAD141 | 0.11 | 0.11 | 0.00 | 2.19 |
| fabAZ | r3HAD160 | 0.09 | 0.09 | 0.00 | 0.00 |
| fabAZ | r3HAD161 | 0.11 | 0.11 | 0.00 | 0.00 |
| fabAZ | r3HAD180 | 0.00 | 0.00 | 0.00 | 0.00 |
| fabAZ | r3HAD181 | 0.00 | 0.00 | 0.00 | 0.00 |
| fadE | ACOAD1f | 0.00 | 0.00 | 0.00 | 0.00 |
| fadE | ACOAD2f | 0.00 | 0.00 | 0.00 | 0.00 |
| fadE | ACOAD3f | 0.00 | 0.00 | 0.00 | 0.00 |
| fadE | ACOAD4f | 0.00 | 0.00 | 0.00 | 0.00 |
| fadE | ACOAD5f | 0.00 | 0.00 | 0.00 | 0.00 |
| fadE | ACOAD6f | 0.00 | 0.00 | -2.19 | 0.00 |
| fadE | ACOAD7f | 0.00 | 0.00 | 0.00 | 0.00 |
| fadE | ACOAD8f | 0.00 | 0.00 | 0.00 | 0.00 |
