# Supplementary material for: Redirector: Designing Cell Factories by Reconstructing the Metabolic Objective
Source: PLoS Comput Biol. 2013 Jan 17;9(1):e1002882. doi: 10.1371/journal.pcbi.1002882 (PMC3547792; doi:10.1371/journal.pcbi.1002882)
Supplement: Table S6 — Myristoyl-CoA production with set boundary limits. This table shows the production of myristoyl-CoA achieved by constraining all reactions associated with the set of enzymes listed in the first column within the boundaries found by boundary analysis. The percent constraint indicates the percentage of maximum production to which the network was constrained when doing the boundary analysis. For example 100% constraintis what was used in finding the boundaries shown in table S5 column E and F, since those boundaries were found while maintain 100% of maximum production flux. The production values are found by imposing the indicated combinations of boundaries on the iAF1260 model then optimized for minimization of the production of myristoyl-CoA (as done in the OptForce approach). All shown boundary combinations were tested in conjunction with a fadE knockout, without which no production was discovered. (DOCX) [file pcbi.1002882.s008.docx]

| Constraints by Enzyme | 80% Constraint | 98% Constraint | 100% Constraint |
| --- | --- | --- | --- |
| fabBF ΔfadE | 0 | 1.92 | 2.19 |
| fabBF ΔfadE tesA | 0 | 1.92 | 2.19 |
| fabBF ΔfadE folD | 0 | 1.92 | 2.19 |
| fabBF ΔfadE fadK or fadD | 0 | 1.92 | 2.19 |
| fabBF ΔfadE acnAB | 0 | 1.92 | 2.19 |
| fabBF ΔfadE aceEF+lpd | 0 | 1.92 | 2.19 |
| fabBF ΔfadE accABCD | 0 | 1.92 | 2.19 |
| fabAZ ΔfadE | 0 | 1.92 | 2.19 |
| fabAZ ΔfadE tesA | 0 | 1.92 | 2.19 |
| fabAZ ΔfadE folD | 0 | 1.92 | 2.19 |
| fabAZ ΔfadE fadK or fadD | 0 | 1.92 | 2.19 |
| fabAZ ΔfadE acnAB | 0 | 1.92 | 2.19 |
| fabAZ ΔfadE aceEF+lpd | 0 | 1.92 | 2.19 |
| fabAZ ΔfadE accABCD | 0 | 1.92 | 2.19 |
| fabG ΔfadE | 0 | 1.92 | 2.19 |
| fabG ΔfadE tesA | 0 | 1.92 | 2.19 |
| fabG ΔfadE folD | 0 | 1.92 | 2.19 |
| fabG ΔfadE fadK or fadD | 0 | 1.92 | 2.19 |
| fabG ΔfadE acnAB | 0 | 1.92 | 2.19 |
| fabG ΔfadE aceEF+lpd | 0 | 1.92 | 2.19 |
| fabG ΔfadE accABCD | 0 | 1.92 | 2.19 |
| fabAZ fabG ΔfadE | 0 | 1.92 | 2.19 |
| fabBF fabG ΔfadE | 0 | 1.92 | 2.19 |
| fabBF fabAZ ΔfadE | 0 | 1.92 | 2.19 |
