# Supplementary material for: Redirector: Designing Cell Factories by Reconstructing the Metabolic Objective
Source: PLoS Comput Biol. 2013 Jan 17;9(1):e1002882. doi: 10.1371/journal.pcbi.1002882 (PMC3547792; doi:10.1371/journal.pcbi.1002882)
Supplement: Table S7 — Experimentally proven overproduction targets from Redirector. Redirector targets (target column) found for overproduction of different products (product column) are shown along with the base strain alterations needed to achieve the production experimentally and percent of the original production achieved. If no wild type strain is shown wild type production levels were too low to be a useful comparison (less than one percent of base line being used). Production levels are given as a percent of the original production of the strain being metabolically altered. Percentages were calculated by look at the ratio of total mg/L produced. (DOCX) [file pcbi.1002882.s009.docx]

| Strain | Targets | Product | Percent Of Original | Reference |
| --- | --- | --- | --- | --- |
| 4CL,CHS,CHI | acc bpl | Naringenin | 100.0 | Fowler,Koffas (2009) |
| 4CL,CHS,CHI | ΔsdhA, acc, bpl | Naringenin | 147.8 | Fowler,Koffas (2009) |
| 4CL,CHS,CHI | ΔglyA, acc, bpl | Naringenin | 129.0 | Fowler,Koffas (2009) |
| 4CL,CHS,CHI | none | Naringenin | 100.0 | Xu,Koffas (2011) |
| 4CL,CHS,CHI | ΔsucC | Naringenin | 122.8 | Xu,Koffas (2011) |
| 4CL,CHS,CHI | ΔfumB | Naringenin | 114.6 | Xu,Koffas (2011) |
| 4CL,CHS,CHI | ΔfumC | Naringenin | 131.0 | Xu,Koffas (2011) |
| 4CL,CHS,CHI | acc | Naringenin | 175.4 | Xu,Koffas (2011) |
| 4CL,CHS,CHI | acc, pgk | Naringenin | 210.5 | Xu,Koffas (2011) |
| 4CL,CHS,CHI | acc, gapD | Naringenin | 198.8 | Xu,Koffas (2011) |
| 4CL,CHS,CHI | acc, pdh | Naringenin | 204.7 | Xu,Koffas (2011) |
| 4CL,CHS,CHI |  | Naringenin | 100.0 | Leonard, Koffas (2008) |
| 4CL,CHS,CHI | cerunlinen  (-fabBF) | Naringenin | 476.2 | Leonard, Koffas (2008) |
| TAL,4CL,CHS,CHI |  | Naringenin | 100.0 | Santos, Stephanopoulos (2011) |
| TAL,4CL,CHS,CHI | cerunlinen  (-fabBF) | Naringenin | 289.7 | Santos, Stephanopoulos (2011) |
| tesA' |  | fatty acids | 100.0 | Davids, Cronan (2000) |
| tesA' | acc | fatty acids | 649.5 | Davids, Cronan (2000) |
| wt |  | C14:0 | 100.0 | Clark, Cronnan (1983) |
|  | fabA | C14:0 | 556.5 | Clark, Cronnan (1983) |
| tesA' |  | fatty acids | 100.0 | Steen, Keasling (2010) |
| tesA' | ΔfadD | fatty acids | 233.3 | Steen, Keasling (2010) |
| tesA' | ΔfadE | fatty acids | 366.7 | Steen, Keasling (2010) |
| atf | ΔfadE | FAEE | 100.0 | Steen, Keasling (2010) |
| atf | tesA', fadD | FAEE | 300.0 | Steen, Keasling (2010) |
| atf | ΔfadE, tesA', fadD | FAEE | 4900.0 | Steen, Keasling (2010) |
