# Supplementary material for: Redirector: Designing Cell Factories by Reconstructing the Metabolic Objective
Source: PLoS Comput Biol. 2013 Jan 17;9(1):e1002882. doi: 10.1371/journal.pcbi.1002882 (PMC3547792; doi:10.1371/journal.pcbi.1002882)
Supplement: Table S8 — Redirector example design update and production. Uptake and export reactions fluxes comparing the flux distribution (optimal biomass), production flux distribution (optimal C14:0-CoA production) and the design used for Figure 1 found by the Redirector framework. To enhance numerical uptake numbers are kept near 10, all fluxes in this work can be projected maintain the same ratio to glucose and O2 uptake, if experimental conditions allow for greater uptake. For all optimizations a minimum of 20% of maximum biomass is maintained. Reactions are identifier names from the iAF1260 model are used. Units used in actual calculations are reduced by a factor of 10 to reduce numerical instability. (DOCX) [file pcbi.1002882.s010.docx]

| Reaction ID | Description | Natural | Production | Redirector | Lower | Upper |
| --- | --- | --- | --- | --- | --- | --- |
| ATPM | ATP maintenance | 8.39 | 8.39 | 8.39 | 8.39 | None |
| EX_o2(e) | O2 uptake | 18.50 | 6.35 | 18.50 | 18.50 | None |
| EX_glc(e) | D-Glucose uptake | 8.00 | 8.00 | 8.00 | 8.00 | None |
| Biomass | E coli biomass | 0.736 | 0.147 | 0.147 | 0.00 | None |
| EX_C14n0_CoA | C14:0-CoA Export | 0.00 | 1.91 | 1.54 | 0.00 | None |
