# Supplementary material for: Redirector: Designing Cell Factories by Reconstructing the Metabolic Objective
Source: PLoS Comput Biol. 2013 Jan 17;9(1):e1002882. doi: 10.1371/journal.pcbi.1002882 (PMC3547792; doi:10.1371/journal.pcbi.1002882)
Supplement: Text S1 — Supplementary methods. The text in this section provides additional information on the construction of the Redirector framework. First is a description of the construction of the redirection function. Second, the construction and usage of inclusion and exclusion variables is explained. It is then shown how these two elements are brought together into a Bilevel MILP formulation. Several methods for constructing redirection coefficient libraries are presented along with the cases in which each would be useful. Next, the iterative local search process used in the framework is presented, showing the progressive search method and how it is used. Finally, a list of variables used in the Redirector framework, and their importance, is given followed by some directions on best practices in using the Redirector approach. Supplementary results. This section includes a graph and discussion of Redirector optimization search times. A contrast of Redirector and flux boundary methods for production of fatty acids is examined. Finally, there is discussion of experimental validation of some of our targets. (DOCX) [file pcbi.1002882.s012.docx]

Supporting Information:

Redirector: Designing Cell Factories by Reconstructing the Metabolic Objective

Table of Contents

[Redirector Search Times 2](#_Toc340747394)

[Construction of Redirection Function for Optimization 2](#_Toc340747395)

[How Inclusion and Exclusion Variables Enable Conversion to MILP 3](#_Toc340747396)

[Bilevel Optimization Problem 3](#_Toc340747397)

[Full Bilevel MILP Formulation 4](#_Toc340747398)

[Redirection Coefficient Library 6](#_Toc340747399)

[Redirection Iterative Local Search Algorithm, Using a Progressive Growth Parameter 7](#_Toc340747400)

[Redirector Variable Descriptions 8](#_Toc340747401)

[Running Redirector 9](#_Toc340747402)

[Fatty Acid Production Using Flux Constraints 9](#_Toc340747403)

[Experimental Validation 11](#_Toc340747404)

# Supplementary Methods

## Construction of Redirection Function for Optimization

In Redirector each enzyme and metabolic change pairing is associated with a binary control variable determining if the associated reaction fluxes and redirection coefficient are included in the redirection function, as the result of a metabolic engineering change. Modeling the relationship between enzymes and reactions gives the ability to optimize complex interactions of metabolic changes in pathways where enzymes are responsible for catalyzing many reactions. Our approach builds on the GDLS method, which introduced the use of gene-protein reaction (GPR) matrices [[40](#_ENREF_40)] for relating gene knockout targets to reactions in the network. The implementation of objective control, allowing for varying up- and down-regulations of chosen targets, requires a unique solution to relate enzymes to the associated reactions. In this work we focus on enzyme targets due to the computational gains from avoiding explicitly modeling the Boolean relationships between genes and enzymes. We build the enzyme selection and enzyme reaction relations for determining which reaction fluxes are in the redirection function of the system objective as follows:

$$\begin{matrix} v_{j}-w_{j}^{l}-u_{j}^{l} & \forall j\in J,l\in L & [S1] \\ y_{c}^{l}'G_{cj}a_{j}\leq w_{j}^{l}\leq y_{c}^{l}'G_{cj}a_{j} & \forall l\in L,c\in C,j\in J & [S2] \\ (1-y_{c}^{l}{'G}_{cj})a_{j}\leq u_{j}^{l}\leq(1-y_{c}^{l}'G_{cj})b_{j} & \forall l\in L,c\in C,j\in J & [S3] \end{matrix}$$

To make the link between fluxes and the redirection function*,* we set reaction flux *v_j_* equal to the sum of the objective inclusion variable *w_j_^l^*, which appears in the redirection function, and the objective exclusion variable *u_j_^l^*. The objective exclusion variable *u_j_^l^* is necessary to construct the MILP formulation of the bilevel problem. The binary control variable *y_c_^l^* controls whether or not the fluxes of the reactions associated with an enzyme *c* are to be included in the redirection function. If *y_c_^l^*=1 the reaction fluxes associated with enzyme *c* and redirection coefficients for metabolic change identifier *l* are included in the redirection function. Conversely if y_c_^l^=0 those fluxes and redirection coefficients are excluded. The enzyme reaction relationship is enforced through the binary matrix *G_cj_*. *G_cj_* =1 if enzyme *c* catalyzes reaction *j* and *G_cj_* =0 otherwise. Using *G_cj_* we can ensure that if a binary control variable is active (*y_c_^l^* =1), only the fluxes of reactions catalyzed by c will be included (*y_c_^l^G_cj_* = 1). For an included reaction, *w_j_^l^* = *v_j_* and *u_j_^l^* =0. Otherwise (*y_c_^l^G_cj_* = 0) and *v_j_* is not included in the redirection function as a result of this metabolic change. If multiple binary variables are allowed to be active for the same enzyme this corresponds to allowing the sum of multiple redirection coefficients for each reaction to be included in the redirection function. Thereby the effect of an enzyme can be tuned, as stated previously. The values *a_j_* and *b_j_* are the lower and upper bounds respectively for the flux v_j_. Each enzyme *c* is a member of the set of allowed enzyme targets C. The set of available enzyme targets can be reduced for faster solving.

## How Inclusion and Exclusion Variables Enable Conversion to MILP

To transform the bilevel optimization formulation into a single level MILP problem, we must construct the relationship between the binary variables of the outer problem (described in the main text) and flux variables of the inner problem in a particular manner. The key to making the bilevel problem into a single level MILP is to construct an alternate form of the inner, FBA optimization (also referred to as the primal problem) that is called the dual problem. To construct the dual problem constraints on the flux variables are transformed into penalties in the objective. The penalties are constructed using new variables called dual variables which form large negative contributions to the objective when the constraints on the flux variables are violated. The contribution of any flux variable to the objective is transformed into a set of constraints which ensures penalties to the objective from the dual variables are constrained. From this process a dual objective is formed. Minimizing the dual objective results in the same optimal solution as maximizing the original FBA objective (primal problem). By setting the primal and dual forms of the inner problem equal, it can be insured that the flux values in the MILP will be constrained to only optimal solutions for the FBA objective.

To allow the outer problem to control the inner problem in the bilevel optimization, the binary variables are used to impose new constraints on the fluxes and dual variables. The primal and dual constraints act oppositely; if a primal variable is constrained to zero then the corresponding dual variable is completely free. Inversely if when a primal variable is unconstrained the dual variable is zero. In this way we can use the binary variables to set primal variables to be free or to be zero. This is why using inclusion and exclusion variables is a good method for allowing reaction fluxes to be included into or excluded from the redirection function. The inclusion and exclusion variables can each be limited to zero or unconstrained by the associated binary variable. Equation 1 in the main text, establishes the relationship between the three variables v, *w* and *u*. Using *u* allows equations 2 and 3 to be formulated such that binary variables can set w and u to zero using binary variables in the primal and dual equality.

## Bilevel Optimization Problem

Objective control and the redirection coefficients are brought together in a bilevel optimization to find an optimal set of enzyme targets and metabolic changes that will increase the production of a metabolite of interest. Once formulated into a bilevel problem, the optimization can be solved as a single MILP. The inner problem is made up of the objective control Z^system^ objective with the inclusion of fluxes in the redirection function being controlled as described above by the binary control variables. These binary control variables are the only link between the inner and outer problem. They are optimized to effectively build the system objective of the inner problem so that flux is redirected to the production objective.

$$\begin{matrix} max & Z^{production}=v_{production} & & & [4] \\ subject to & \sum_{lc} y_{c}^{l}\leq k & & & [5] \\ & \sum_{l} y_{c}^{l}\leq s & & \forall c\in C & [6] \\ & y_{c}^{l}\in(0,1) & & \forall l\in L,c\in C & \\ & max & Z^{system}=\gamma v_{biomass}+\sum_{jl} \beta_{j}^{l}'w_{j}^{l} & & [1] \\ & subject to & \sum_{j} S_{ij}v_{j}=0 & \forall i\in I & [7] \\ & & v_{j}-w_{j}^{l}-u_{j}^{l}=0 & \forall j\in J,l\in L & \left[ S1 \right] \\ & & y_{c}^{l}G_{cj}a_{j}\leq w_{j}^{l}\leq y_{c}^{l}'G_{cj}a_{j} & \forall l\in L,c\in C & [S2] \\ & & (1-y_{c}^{l}{'G}_{cj})a_{j}\leq u_{j}^{l}\leq(1-y_{c}^{l}'G_{cj})b_{j} & \forall l\in L,c\in C & [S3] \end{matrix}$$

The problem is optimized for the outer objective Z^production^ which is equal to the production flux v_production_. The inner problem objective is the system objective. The number of enzyme targets *c* and different metabolic changes *l* chosen in one iteration is constrained by limiting the sum of the binary control variables which are being activated ( y_c_^l^=1) to be less than *k*, the local neighborhood search size. The optimization is able to finely tune the optimal impact (sum of redirection coefficients) on an enzyme target by activating multiple binary control values for the same target. To constrain the search space allocated to tuning metabolic changes, the number of simultaneously active binary control variables, for a single enzyme *c*, is limited by *s*, referred to as the coefficient tuning variable. For example, if k=3 and s=2 in one iteration, it would be possible for 3 separate enzymes to be targeted by a single change. Alternatively, it would be possible for one enzyme to be tuned by combining 2 redirection coefficients and one other enzyme to be targeted by a single redirection coefficient.

## Full Bilevel MILP Formulation

Here we describe how the Objective Control bilevel optimization can be reformulated into a single MILP optimization. Previously we presented the variables (u and w) and equations (eq. S1) which form objective control as separate from those that form the standard formulation of FBA linear optimization problem (eq. 7). To construct the MILP problem to solve the bilevel optimization problem in the Redirector method, we reformulate the inner problem back into a standard linear optimization (Sv=0). We do this by unifying all equations into a single matrix S^*^ and all variables into a single vector *v*^*^. Using the standard formulation of the linear problem allows us to directly construct a corresponding standard dual formulation and use primal/dual equality to formulate the MILP from the bilevel problem. Using these standard formulations makes constructing a dual of the inner problem and the final MILP much easier to follow and illustrates the fact that the objective control formulation of the FBA problem is still very much a linear optimization.

The following is a list of new variables and indexes used in the reformatted primal problem.

$$\begin{matrix} v_{m}^{*}=v_{j} & \forall j\in J,m\in M^{v} \\ v_{m}^{*}=w_{j}^{l} & \forall j\in J,l\in L,m\in M^{w} \\ v_{m}^{*}=u_{j}^{l} & \forall j\in J,l\in L,m\in M^{u} \\ {{f'}_{m}}^{*}=\gamma& j=biomass,m=biomass \\ {{f'}_{m}}^{*}=\beta_{j}^{l}' & m\in M^{w} \\ S_{pm}^{*}=S_{ij} & \forall i\in I,j\in J,p\in P^{i},m\in M^{v} \\ S_{pm}^{*}v_{m}^{*}=v_{j}-w_{j}^{l}-u_{j}^{l} & \forall j\in J,l\in L,p\in P^{ii} \end{matrix}$$

The main reformulation is bringing all the dependent variables *v*, *u* and *w* (described in the sections above) together into a new a single set of variables v^*^. The new master index *m* of *v*^*^, joins the combinations of index *j* of *v* with the combination of indexes *j* and *l*, for *u* and *w*. For clarity the total set of M is divided into three regions such that the original variables can be referenced easily as part of v^*^. Correspondingly, v is indexed by M^v^, u is indexed by M^u^ and w is indexed by M^w^. Similarly the new linear optimization matrix S^*^ encompasses the mass conservation limitation of stoichiometric matrix S and equations for the objective control relationships of *v*, *u* and *w*. The new matrix *S*^*^ is indexed by a new row index *p* which combines the metabolite indexes and indexes for the v, u and w constraints, as well as the new column index *m*. Finally, to complete the conversion to the general form of the linear optimization, the vector f^*^ combines the coefficient ϒ for the biomass flux v_biomass_ with the redirection coefficients β for the linking variables w.

The reformatted primal formulation of the objective control problem is now standard LP formulation plus boolean constraints using the enzyme reaction matrix G, as follows.

$$\begin{matrix} max & \sum_{m} f_{m}^{*}v_{m}^{*} & \\ subject to & \sum_{m} S_{pm}^{*}v_{m}^{*}=0 & p\in P^{i,ii} \\ & y_{c}^{l}'G_{cj}a_{j}\leq v_{m}^{*}\leq y_{c}^{l}'G_{cj}b_{j} & \forall l\in L,c\in C,m\in M^{w} \\ & (1-y_{c}^{l}{'G}_{cj})a_{j}\leq v_{m}^{*}\leq(1-y_{c}^{l}'G_{cj})b_{j} & \forall l\in L,c\in C,m\in M^{u} \end{matrix}$$

The corresponding dual form of the problem is.

$$\begin{matrix} max & \sum_{m} {\nu_{m}^{*}b_{m}^{*}-\mu}_{m}^{*}a_{m}^{*} & \\ subject to & f_{m}^{*}-\sum_{p} \lambda_{p}S_{pm}-{\nu_{m}^{*}-\mu}_{m}^{*}-\xi_{m} & m\in M^{v,u,w} \\ & -\left( 1-y_{c}^{l}{'G}_{cm} \right)D\leq\xi_{m}\leq(1-y_{c}^{l}'G_{cm})D & \forall l\in L,c\in C,m\in M^{w} \\ & -y_{c}^{l}'G_{cm}D\leq\xi_{m}\leq y_{c}^{l}'G_{cm}D & \forall l\in L,c\in C,m\in M^{u} \end{matrix}$$

This formulation of the dual is very similar to the formulation of the boolean constraints on fluxes used in GDLS. The main difference is rather than constraining fluxes variables directly, linking and unlinking variables are controlled by the boolean variables. The same boolean constraints that controls when v^*^ is free or equal to zero also controls when the slack variable $\xi$ is free or constrained to zero. The limit variable D is a large number allowing $\xi$ to be unconstrained by this value given the other uptake limitations unless D is multiplied by zero. Typically 1000 is sufficiently large value for D with uptake constraints used on the model in this analysis. The boolean relation is reversed for values of v^*^ corresponding to the linking variable w (M^w^) and those corresponding to the unlinking variable u (M^u^) in both the primal and dual formulations. The result is that when v^*^ is zero for one set of indexes $\xi$ is free for that set of indexes, and vice versa. This is due to the fact that when a primal variable is unconstrained, the corresponding dual penalty is zero and when a primal variable is constrained to zero the corresponding dual penalty is free to be as large as possible.

Bringing these primal and dual formulations together, while setting their objectives to be equal, yields the single level MILP of the redirector method.

$$\begin{matrix} max & Z^{production}=v_{production} & \\ subject to & f_{m}^{*}-\sum_{p} \lambda_{p}S_{pm}-{\nu_{m}^{*}-\mu}_{m}^{*}-\xi_{m} & m\in M^{v,u,w} \\ & \sum_{m} f_{m}^{*}v_{m}^{*}=\sum_{m} {\nu_{m}^{*}b_{m}^{*}-\mu}_{m}^{*}a_{m}^{*} & \\ & \sum_{m} S_{pm}^{*}v_{m}^{*}=0 & p\in P^{i,ii} \\ & f_{m}^{*}-\sum_{p} \lambda_{p}S_{pm}-{\nu_{m}^{*}-\mu}_{m}^{*}-\xi_{m} & m\in M^{v,u,w} \\ & y_{c}^{l}'G_{cm}a_{m}\leq v_{m}^{*}\leq y_{c}^{l}'G_{cm}b_{m} & \forall l\in L,c\in C,m\in M^{w} \\ & (1-y_{c}^{l}{'G}_{cm})a_{m}\leq v_{m}^{*}\leq(1-y_{c}^{l}'G_{cm})b_{m} & \forall l\in L,c\in C,m\in M^{u} \\ & (1-y_{c}^{l}{'G}_{cm})a_{j}\leq\xi_{m}\leq(1-y_{c}^{l}'G_{cm})b_{m} & \forall l\in L,c\in C,m\in M^{w} \\ & y_{c}^{l}'G_{cm}a_{m}\leq\xi_{m}\leq y_{c}^{l}'G_{cm}b_{m} & \forall l\in L,c\in C,m\in M^{u} \end{matrix}$$

## Redirection Coefficient Library

$$\begin{matrix} flat & \beta_{j}^{l}\in(1,-1) & \forall j\in J \\ power & \beta_{j}^{l}\in{(2}^{n},-2^{n}) & \forall j\in J,n\leq0 \\ sensitivity & \beta_{j}^{l}=\frac{dZ}{dv_{j}} & \forall j\in J \end{matrix}$$

The first approach is to build a redirection coefficient library with the same set of redirection coefficients for every reaction. This approach has the advantage that all reactions and enzymes are equally accounted for and no prior analysis of the network is required. The simplest and most straight forward such redirection coefficient library is one in which every flux is associated to redirection coefficients of equal positive and negative weight (β= +/-1.0). We call this the “flat redirection coefficient library”, as it allows for metabolic changes with equal positive and negative effects. However, this flat redirection coefficient library can easily be expanded to allow for a selection of different magnitudes of metabolic engineering changes from which the optimal choice can be made. This is done by including a larger selection of redirection coefficients and the option for multiple redirection coefficients to be active for each enzyme. To expand the flat redirection coefficient library in this way we develop a “power series redirection coefficient library”, which is composed of a binary power series of redirection coefficients, β= +/-2^n^ with n≤0 (e.g. every reaction has associated redirection coefficients of +/-1, +/-0.5, +/-0.25 and so on). This also allows for a summation of multiple redirection coefficients for one reaction to reach any effective redirection coefficient with a resolution of 2^min(n)^ and a maximum absolute value of all the redirection coefficients summed. Changing the number of values of *n* used in the power series library allows us to tailor the number of redirection coefficients to the computational power available. Computational power needed and n are directly linked by the number of binary control variables needed to allow for each possible metabolic engineering change identifier *l* in the power series library. The result is that the power series redirection coefficient library allows the discovery of the optimum balance of metabolic engineering changes by choosing from a number of coefficients for each reaction added to the system objective and the fine tuning of the incentive on each of those reactions.

We also developed a “sensitivity redirection coefficient library” based on sensitivity of the growth and production objectives to reaction flux changes. This method takes into account how each metabolic change will work for or against both growth and the metabolite production in determining the strength of the associated redirection coefficients. The sensitivity redirection coefficients are calculated as: β_j_^positive^ = max(*dZ^optimal^/dv_j_* ) > δ^sense^ and β_j_^negative^ = min(*dZ^optimal^/dv_j_* ) < -δ^sense^, which describe the maximum positive and negative effect that changing a flux *v_j_* will have on the optimized value of Z. To find redirection coefficients which counteract the growth function we use Z=*-v^biomass^*, where v^biomass^ is the flux towards biomass. Redirection coefficients that contribute to the production objective are found using Z= v^production^, where v^production^ is the flux towards the metabolite of interest. For the sake of reducing search space and improving numerical stability during the optimization, we ignore sensitivity redirection coefficients with an absolute value less than δ^sense^ = 0.01. The sensitivity coefficients are computed over the range *a_j_*<*v_j_*<*b_j_* where *a_j_* and *b_j_* are the minimum and maximum possible values of flux *v_j_*. The sensitivity redirection coefficient library provides a normalized value for redirection coefficients relative to the magnitude of each flux and the objective for which it was computed. Enzyme targets that have no associated redirection coefficients are not included in the optimization. This method reduces the number of binary control variables needed in the bilevel optimization, because it only produces redirection coefficients for fluxes that affect either the growth function or production objective directly, reducing the computational resources required by the Redirector framework.

## Redirection Iterative Local Search Algorithm, Using a Progressive Growth Parameter

Here we describe the logic of how Redirector performs an iterative local search for target discovery using the progressive growth parameter. After completing the bilevel optimization the Redirector framework checks if any new targets have been discovered as well as the value of the production objective, at this iteration. It is then determined if the value of the progressive growth coefficient ϒ needs to be increased to discover more targets, or if no more targets can be discovered and the search has ended. To do this we use a cut off for near maximum production for determining when to increase the progressive growth parameter. This near maximum production is defined as 80% of the maximum possible value of the production objective while maintaining our requirement for 20% of maximum growth. If the production objective is near its maximum value then the redirection function is no longer being driven to discover new targets and the value of ϒ is increased to ϒ^new^ as shown in eq. 11 in the main text. Alternatively, if new targets are discovered and the production objective is not near maximum, then the value of the progressive growth parameter is still driving discovery and the search continues with the same value of ϒ. Finally, if there are no new targets and design production is low (<20% max(Z^production^)) we conclude that there are no designs in this search range that can overcome that ϒ level. Usually the design discovered in the step before this last scenario is the final design discovered, because ϒ is only ever increased slightly above the effective value of the redirection function. However, the method double checks that this is the maximal design by reducing the value of ϒ. The value of the progressive growth parameter is reduced to slightly less than the calculated effective value of the redirection function. This is done by subtracting instead of adding δ in the formulation of ϒ^new^ above. Then the optimization is rerun and the method checks that the previous design is sufficient to overcome this value of the growth function. If instead new targets are once again discovered to overcome this value of the growth function then the search continues. Once this confirmation is complete the search ends.

During iterative optimization, binary variables that are inactive in the previous iteration continue to use the inclusion formulation, shown above. Conversely active binary variables from the previous iteration are made to be “active” by default (*y_c_^l^* =0). This is done by switching the inclusion constraints such that *y_c_^l^G_cj_* becomes (1- *y_c_^l^G_cj_* ) in the upper and lower bounds of equation 2 and (1- *y_c_^l^G_cj_* ) becomes *y_c_^l^G_cj_* for the bounds of equation 3. This reverses the relationship between binary variables and both inclusion and exclusion variables for the active targets from the previous iteration.

## Redirector Variable Descriptions

We provide Supporting Information Table S3 to describe the variables used in Redirector. The determination of values of redirection coefficients $(\beta)$ is done before the search. We present three different methods for choosing values of $(\beta)$ in detail in methods Supporting Information section Redirection Coefficient Library. The variable *i* tracks the current number of iterations that have passed in the current iterative local search. The parameters *k* and *s* control the search space of targets for metabolic alterations. The best value of k is determined by the number of targets being searched and the computational power available, the smaller the target size and more computational power the larger a value of k one can use. The method for determining the value of the progressive growth parameter ($\gamma$) is described in the main article Methods section Progressive Target Discovery and depends directly on the previously discovered set of targets and δ^progress^. The variable δ^progress^ is a small number, the specific value of which is of little importance as long as it remains significantly smaller (2 orders of magnitude is sufficient) than the value of the progressive growth parameter ($\gamma$). Table S4 shows that varying the value of δ^progress^, over 4 orders of magnitude, has no effect on the progress or results of the redirector search but it is necessary to allow the progressive target discovery method to function.

## Running Redirector

The Redirector framework is built with a flexible robust structure working with the bilevel FBA to maximize applicability and design options for any metabolic model and optimization phenotype desired. Optimization searches can be tailored to suit the particular design parameters of the user. For maximal design coverage we recommend using various search sizes with the flat and sensitivity coefficient libraries for a number of iterations, maximizing the use of computational resources. The use of both flat and sensitivity coefficient libraries combine the advantages of both libraries and gives largest possible solution space to draw from. If this results in a large number of targets and designs, more than can be optimized in the first round of metabolic engineering, then dependency network analysis can be used to find the core targets for optimizations which can be used as a starting point to build upon in later rounds of engineering. When computational power is a concern, smaller search sizes can be used along with the sensitivity library to reduce search times. The power series redirection coefficient library with multiple possible engineering changes per enzyme group can be used to best balance the relative contribution to the production from each reaction, though this is computationally intensive. Another method that can be used is to run an optimization with flat or sensitivity coefficients then use the targets found in that run to “pre-load” targets into a power series coefficient optimization. This pre-loaded powers series library optimization will then take targets from a less computationally intensive flat/sensitivity coefficient optimization and balance those targets with the power series coefficients. Pre-loading an optimization can be done with any set of targets whether from a previous optimization or from experimental evidence. A pre-loaded optimization can still add and remove targets as it normally would to find the optimal set of targets available at that neighborhood size and redirection coefficient library.

# Supplementary Results

## Redirector Search Times

The search times in Figure S1 show the unpredictability of the time needed to optimize a bilevel framework. While there is a trend for smaller search neighborhoods to require less time especially in the case of the flat control library and k=1 sensitivity library, it is not a rule by any means. In fact we observe k=4 and k=5 for the flat control searches are unable to find any more designs after iteration I=3 and I=6 respectively. In contrast k = 6 continues to find solutions for all 10 iterations even though the initial k=6 search takes longer than k=5 to reach I=6. Also it can be observed that in the sensitivity control example search sizes k=2 and k=5 both take quite a while to find initial targets but then quickly find additional targets after I=1.

These search times illustrate the importance of flexible search parameters and searching with different values of *k*. As the value of *k* increases it is possible to discovering designs that require several simultaneously control targets while others may necessitate time to bypass bilevel optimization difficulties where many solutions are equally valid. However as the value of *k* increases the search space becomes exponentially larger and at some point no solutions are found in reasonable time.

## Fatty Acid Production Using Flux Constraints

To provide a comparison for Redirector approach, we performed an analysis using a flux boundary based model of metabolic alterations targeting enzymes, to optimize the production of fatty acids (myristoyl-CoA). In doing so we replicated the fundamental methods of OptForce (the creation of MUST and FORCE sets). We focused on a small group of targets to allow us to replicate the functional principles of the method without needing to reconstruct the entire framework. To build our set of test targets we used the set of experimentally proven enzymes (accABCD, fadE, fabA Z, fadD and tesA ), the remaining fatty acid biosynthesis enzymes (fabG and fabBF) and a few likely enzymes found by the Redirector method (aceEF and lpd, acnAB, folD). Correspondingly, the list of reactions tested was all those mediated by these enzymes, as indicated in the iAF1260 model. To carry out this comparison, we first performed a boundary analysis, finding the flux bounds associated with our two objectives (biomass and myristoyl-CoA production) separately and comparing them. Then we tested all combinations of these boundaries (grouped by enzyme) to see if we could achieve production of myristoyl-CoA. The production level was found, as each combination of bounds was tested as limits in the iAF1260 FBA model of E. coli, while optimizing for the minimization of the production of myristoyl-CoA (as done for the discovery of FORCE sets in OptForce).

Our boundary analysis was performed by comparing viable flux space (range of possible values of each reaction flux), while maintaining near optimal values for each of our two competing objectives individually, biomass vs. myristoyl-CoA with production of at least 80%, 90%, 98% and 100% of maximum values. The range of values is found by minimizing and maximizing each flux respectively, while maintaining the requisite percentage of the requisite flux towards the objective of interest (biomass or myristoyol-CoA). Reactions are considered potential targets if their fluxes have non-overlapping boundaries when comparing between the biomass and production boundary sets. Reactions in which the production boundaries are higher than the biomass boundaries are considered targets for up-regulation and conversely if production boundaries are lower than those for biomass the reaction is a target for down-regulation or being knocked-out. The results of this analysis are shown in separate excel Supporting Information Table S9 and a representative set of flux boundaries is shown in Table S5. Specifically Table S5 shows the boundaries found when maintaining 100% production and biomass, as it is the strictest case and misses the fewest potential targets due to overlapping values in the two flux boundary sets.

Table S5 illustrates one of the fundamental weaknesses of modeling metabolic alterations using flux constraints. It can be observed that reactions catalyzed by single enzymes like *accABCD* show good separation between their flux boundaries for optimal biomass compared to optimal production and indicates that the flux should be altered in the expected direction (increased). However the reactions catalyzed by fabAZ produce a more complex situation. The reactions catalyzed by fabAZ which result in the production of short chain fatty acids (C6 to C10) all have consistently higher flux boundaries when maintaining high production compared to that of biomass, indicating viable targets for being up-regulated. The reactions for producing medium chain saturated and unsaturated fatty acids (C12:0, C12:1, C14:0 and C14:0) have overlapping boundaries between the two sets. Reactions producing larger fatty acid products (C16:0, C16:1, C18:0 and C18:1) show lower boundaries in the production set indicating they are targets for down-regulation or knockouts. This means reactions catalyzed by enzyme group fabAZ fall into the up-regulation, invalid metabolic alteration target, and down regulation/knockout target groups. Furthermore the same problem was observed for the other fatty acid biosynthesis genes. Including further information (such as metabolomics data) could help restrict the biomass flux boundaries. However, reactions mediated by the same fatty acid biosynthesis enzymes change in fundamentally different directions, when comparing flux bounds between biomass and overproduction networks. While MFA data can reduce overlapping problems with overlapping flux bounds, mapping enzyme changes to reactions would remain a problem.

To test the capacity of these boundaries to produce myristoyl-CoA we tested all combinations of boundaries grouped by enzyme for percentage of production. Resulting production levels of myristoyl-CoA are shown in Table S6. We were able to achieve production of myristoyl-CoA only when we included those boundaries which forced fluxes in different directions for reactions catalyzed by the same enzyme (fabAZ, fabBF and fabG). We also tried constructing boundaries for the sum of all fluxes from reactions catalyzed by the same enzyme, for example the sum of fluxes for all 10 reactions catalyzed by *fabAZ* was given a constraint. This replicated the method for creating a MUST set indicating the up-regulation of all 10 of these reaction as a group. However, putting a boundary on the sum of the fluxes allowed the involved fluxes a great deal of freedom. As a result all of our attempts to use boundaries on the sum of fluxes for reactions catalyzed by the same enzyme failed to yield any production.

Looking at the Table S5, we observer that no combination of flux bounds for 80% myristoyl-CoA production when applied as metabolic alterations, achieved any level of myristoyl-CoA production. Using the flux bounds for 98% and 100% production did yield production of myristoyl-CoA. However, production was only achieved when knocking out fadE was paired with constraints for fatty acid biosynthesis enzyme (fabAZ, fabBF or fabG). As previously mentioned these fatty acid biosynthesis enzymes mediate reactions with boundaries that shift fluxes in different directions for reactions controlled by the same enzyme. We also observed that no additional production was achieved by the addition of any further enzyme constraints, even those for experimentally proven metabolic alteration targets. Due to the fact that there is no improvement in production from the addition of these targets, there is no reason to select any of these experimental proven enzyme targets (accABCD, fadD or tesA). In fact, the OptForce method minimizes the number of targets to only those that are required to achieve production, so it can be assured that none of these experimentally proven targets would be selected.

## Experimental Validation

As a proof of principle we have confirmed several core targets discovered by our framework, by carrying out a small scale experimental analysis.  We have taken a λ-red recombination strain called EcfC1, knocked out the fatty acid β-oxidation gene *fadE*, then inserted two plasmids separately, one plasmid over-expressing *fadD* and *‘tesA* (pZA41-tAfD) with anhydrotetracycline (ATc) induction, and another plasmid over-expressing *fadD, fadR* and *‘tesA*(pZA41-tAfDfR) with ATc induction.  These 4 cell types (EcfC1, EcfC1 ΔfadE, EcfC1 ΔfadE pZA41-tAfD and EcfC1 ΔfadE pZA41-tAfDfR) were grown up in M9 minimal media supplemented with 0.04% acetate as a carbon source, cassamino acids, thiamine, D-biotin and carbenicillin for cells with the β-lactamse cassette or spectinomycin for those cells with spectinomycin resistant plasmids.  These cells were grown to stationary phase over 48 hours with varying levels of ATc induction, then the cells and media were subjected to sonication.  All sonicated cultures were then measured for fatty acid levels with the Free fatty acids, Half-micro test from Roche according to their protocol and corrected for cell density using optical density measured at 600 nm.  The results from this test appear in Supporting Information Figure S2.

The fatty acid levels measured for these cells (Figure S2) show that as we add genetic manipulations to our strain the relative production of fatty acids increases.  Knocking out *fadE* increases production by 25% from the original strain.  Over-expressing *fadD* and *‘tesA,*along with the *fadE* knockout, triple the production of fatty acids from the original strain with maximum ATc induction.  Adding the effects of *fadR* over-expression, at 5 ng/ml ATc, increased the fatty acid production ~325% from the original EcfC1 strain.

The results from Figure S2 support optimization targets from our Redirector predictions.  As predicted by our framework, *fadE*alone can increase fatty acid production.  Since we were optimizing for acyl-CoA our results from redirector predicted that *fadD* and *tesA* would increase production by removing an ACP group from acyl-ACP (*tesA*) and adding a CoA (*fadD*) to the acyl group.  Conveniently, the Roche Half-micro test can measure acyl-CoA directly, capturing increases in acyl-CoA production resulting from over-expressing these genes.  Finally, fadR functions to repress fatty acid β-oxidation genes such as *fadA*, *fadI* and *fadK* while activating fatty acid biosynthesis genes such as *fabA* and *fabB*.  All of these genes are targeted by our optimization and the predicted fatty acid production increases from these changes are borne out by this experiment.
